# Targeting endothelial junctional adhesion molecule-A/ EPAC/ Rap-1 axis as a novel strategy to increase stem cell engraftment in dystrophic muscles

**DOI:** 10.1002/emmm.201302520

**Published:** 2013-12-30

**Authors:** Monica Giannotta, Sara Benedetti, Francesco Saverio Tedesco, Monica Corada, Marianna Trani, Rocco D'Antuono, Queensta Millet, Fabrizio Orsenigo, Beatriz G Gálvez, Giulio Cossu, Elisabetta Dejana

**Affiliations:** 1 FIRC Institute of Molecular Oncology Foundation (IFOM)Milan, Italy; 2 Department of Cell and Developmental Biology, University College LondonLondon, UK; 3 Division of Regenerative Medicine, Stem Cells and Gene Therapy, San Raffaele HospitalMilan, Italy; 4 National Centre for Cardiovascular ResearchMadrid, Spain; 5 Institute of Inflammation and Repair, University of ManchesterManchester, UK; 6 Department of Biosciences, School of Sciences, University of MilanMilan, Italy

**Keywords:** endothelial cells, junctional adhesion molecule-A, muscular dystrophy, stem cell therapies

## Abstract

Muscular dystrophies are severe genetic diseases for which no efficacious therapies exist. Experimental clinical treatments include intra-arterial administration of vessel-associated stem cells, called mesoangioblasts (MABs). However, one of the limitations of this approach is the relatively low number of cells that engraft the diseased tissue, due, at least in part, to the sub-optimal efficiency of extravasation, whose mechanisms for MAB are unknown. Leukocytes emigrate into the inflamed tissues by crossing endothelial cell-to-cell junctions and junctional proteins direct and control leukocyte diapedesis. Here, we identify the endothelial junctional protein JAM-A as a key regulator of MAB extravasation. We show that *JAM-A* gene inactivation and JAM-A blocking antibodies strongly enhance MAB engraftment in dystrophic muscle. In the absence of JAM-A, the exchange factors EPAC-1 and 2 are down-regulated, which prevents the activation of the small GTPase Rap-1. As a consequence, junction tightening is reduced, allowing MAB diapedesis. Notably, pharmacological inhibition of Rap-1 increases MAB engraftment in dystrophic muscle, which results into a significant improvement of muscle function offering a novel strategy for stem cell-based therapies.

## Introduction

Muscular dystrophies are inherited neuromuscular disorders that are characterized by progressive muscle wasting and weakness that lead to a wheelchair confinement and to a heart and/or respiratory failure in the most severe forms (Emery, 2002; Mercuri & Muntoni, 2013). Although several new gene-therapy and cell-therapy strategies are currently under clinical investigation (Partridge, 2011; Arechavala-Gomeza *et al*, 2012; Tedesco & Cossu, 2012; Benedetti *et al*, 2013), to date there are no successful and definitive treatments. Approximately a decade ago, we described a population of myogenic vessel-associated stem/progenitor cells, known as mesoangioblasts (MABs) (De Angelis *et al*, 1999; Minasi *et al*, 2002). When MABs were injected into the femoral artery of different pre-clinical models of muscular dystrophies, amelioration of the dystrophic phenotype was observed (Sampaolesi *et al*, 2003, 2006; Galvez *et al*, 2006; Gargioli *et al*, 2008; Diaz-Manera *et al*, 2010; Tedesco *et al*, 2011). Similar cells were isolated from human post-natal skeletal muscle (human MABs) and they were characterized as a subset of pericytes with myogenic potency *in vitro* and *in vivo* (Dellavalle *et al*, 2007). Based on these pre-clinical studies, a phase I/II clinical trial based upon intra-arterial allogeneic transplantation of MABs is currently ongoing for Duchenne muscular dystrophy (EudraCT no. 2011-000176-33). However, one limitation with this cell therapy is the difficulty of ensuring that a sufficient number of cells reach the damaged areas, especially when they are infused through the vascular route. Additionally, the mechanism by which MABs cross the vessel wall and infiltrate the damaged tissue is still poorly understood.

Leukocyte extravasation can be stimulated by a repertoire of cytokines that are produced by the inflamed tissue, as well as by up-regulation of specific adhesion molecules on the endothelial surface (Butcher, 1991; Nourshargh *et al*, 2010). In a previous study, we showed that the combined pre-treatment of MABs with stromal-derived factor-1 (SDF-1) and tumor necrosis factor-α (TNF-α) enhanced MAB extravasation through endothelial cells *in vitro* and *in vivo* (Galvez *et al*, 2006).

In most cases, leukocyte extravasation occurs through endothelial cell-to-cell junctions (Vestweber, 2002; Woodfin *et al*, 2007). These structures include both adherens and tight junctions that are formed by a complex network of transmembrane adhesive proteins linked inside the cells to cytoskeletal and signaling partners. Among these, platelet endothelial cell adhesion molecule-1 (PECAM-1) and junctional adhesion molecule-A (JAM-A) are the most effective in the modulation of leukocyte diapedesis through endothelial junctions (Nourshargh *et al*, 2006). JAM-A is a small immunoglobulin that is located at endothelial and epithelial cell tight junctions (Martin-Padura *et al*, 1998; Bazzoni & Dejana, 2004; Ebnet *et al*, 2004; Imhof & Aurrand-Lions, 2004), and it appears to have multiple actions by interacting different intracellular partners (Martin-Padura *et al*, 1998; Bazzoni *et al*, 2000; Ebnet *et al*, 2000). Of note, JAM-A becomes concentrated at junctions as soon as cells come into contact, and it promotes the correct assembly of the other members of these junctional structures (Bazzoni & Dejana, 2004; Iden *et al*, 2012). The importance of JAM-A in endothelial and epithelial barrier function has been studied previously (Liu *et al*, 2000; Mandell *et al*, 2005, 2006, 2007). However, the downstream signaling pathways linking JAM-A to the regulation of the junctional tightening are still only partially understood.

In the present study, we show that inhibition of JAM-A expression and/or activity enhances the engraftment of MABs to dystrophic muscle. Moreover, JAM-A inhibition is associated with a strong reduction in Rap-1 activity, which, in normal conditions, is known to maintain junction integrity.

Importantly, these studies led to identify that the chemical inhibition of Rap-1 reproduces the effects of JAM-A abrogation, thus opening the possibility of developing specific drugs to improve stem cell engraftment. These findings introduce a novel strategy of intervention to improve stem cell therapies in dystrophic patients.

## Results

### MAB engraftment into acutely injured skeletal muscle is increased in *JAM-A*-null mice

**Figure 1:**
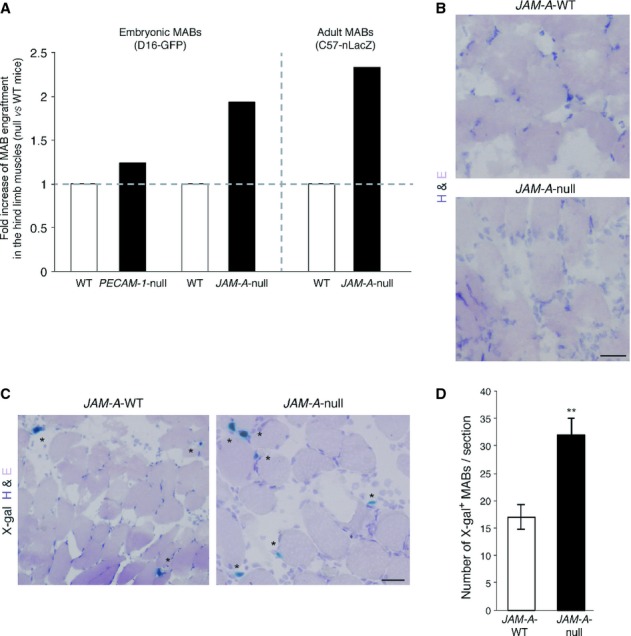
D16-GFP embryonic or C57-nLacZ adult murine mesoangioblasts (MABs) were injected into the right femoral artery of WT, *PECAM-1*-null and *JAM-A*-null age-matched mice (treated with cardiotoxin 24 h before transplantation) as indicated. After 6 h, the hind limb muscles (gastrocnemius, tibialis anterior and quadriceps) were collected and the presence of migrated cells was quantified using qRT-PCR with GFP or nLacZ primers. The RNA levels were normalized using GAPDH. The RNA relative levels for the controls were set to 1, and the ratios for *PECAM-1*-null ( *n* = 7) or *JAM-A*-null ( *n* = 10) *versus* WT ( *n* = 17) are shown for embryonic (left) and adult (right) murine MABs. Fold increases have been extrapolated by data shown in Figure S1A–E.Representative Hematoxilin and Eosin (H&E) staining of *JAM-A*-WT and *JAM-A*-null mice from A after cardiotoxin treatment. Scale bars: 50 μm.Representative cryosections of the gastrocnemius muscle of *JAM-A-*WT and *JAM-A*-null mice stained for with H&E and X-gal. Asterisks indicate donor cells. Scale bars: 50 μm.Quantification of X-gal^+^ MABs in cryosections illustrated in C. ** *P *< **0.05. Data are expressed as means ± s.e.m.

To investigate the involvement of the endothelium in MAB engraftment, we focused on two endothelial junction proteins with established functions in leukocyte diapedesis: JAM-A and PECAM-1 (Bazzoni & Dejana, 2004; Woodfin *et al*, 2007). At first, the *in vivo* migration of MABs from the vessel lumen to the muscle interstitial tissues was assessed in genetically modified JAM-A and PECAM-1 deficient mice ( *JAM-A*-null and *PECAM-1*-null mice, respectively) (Duncan *et al*, 1999; Cera *et al*, 2004). To this end, green fluorescent protein (GFP)-expressing murine embryonic MABs (D16-GFP) (Sampaolesi *et al*, 2003) were injected into the right femoral artery of wild-type (WT), *JAM-A*-null and *PECAM-1*-null age-matched mice after acute muscular injury caused by a previous intra-muscular injection of cardiotoxin. After 6 h, the mice were sacrificed and the hind limb muscles (gastrocnemius, tibialis anterior and quadriceps) were collected and their RNA was extracted. qRT-PCR of GFP expression was used to evaluate the presence of migrated cells. Embryonic MABs migrated significantly more efficiently to the injured muscles of the *JAM-A*-null mice than WT, although they did not show a significant increase in engraftment over WT when injected into the *PECAM-1-*null mice (Fig 1A, on the left of the dashed line and supplementary Fig S1A–D). This suggested a selective and specific role for JAM-A in MAB transmigration across the endothelium.

We then extended these experiments to nuclear LacZ-expressing adult murine MABs (C57-nLacZ) (Diaz-Manera *et al*, 2010), which, at variance with embryonic MABs, have spontaneous myogenic potential (supplementary Fig S2A). qRT-PCR analysis for nLacZ cDNA revealed that adult MABs can also cross the endothelium to reach the cardiotoxin damaged muscle tissue (Fig 1B) with a higher efficiency in the *JAM-A*-null mice compared to the WT mice (Fig 1A, and supplementary Fig S1E).

X-gal staining performed on gastrocnemius muscle sections damaged by cardiotoxin was consistent with qRT-PCR results (Fig 1C and D). As quantified in Fig 1D we measured up to a one-fold increase in X-gal^+^ MABs in the injured muscles of *JAM-A*-null mice compared to WT. These data suggest that impairment of JAM-A expression significantly augments MAB engraftment to the damaged muscle.

### Chronic and acute inhibition of JAM-A enhances MAB engraftment into dystrophic muscle of *Sgca*-null mice

To overcome the variability in the levels of muscle damage induced by cardiotoxin and to study JAM-A role in muscular dystrophy, *JAM-A*-null mice were crossed with *alpha-Sarcoglycan* ( *Sgca)*-null mice. The *Sgca*-null mice are a model for limb-girdle muscular dystrophy type 2D (LGMD2D), caused by mutations in the gene that encodes alpha-sarcoglycan protein (Duclos *et al*, 1998). The resulting double-null mutation was confirmed by PCR genotyping (supplementary Fig S3A; both mouse strains were in a C57/BL6 genetic background). We first compared the muscle histopathology of the *Sgca*-null mice with that of the *Sgca-*null/ *JAM-A*-null mice with hematoxylin and eosin staining of transversal sections of the gastrocnemius muscle. This analysis revealed a similar phenotype that was characterized by several regenerating skeletal muscle fibers with central nuclei and variations in myofiber size (supplementary Fig S3B), which are some of the typical features of dystrophic muscle.

**Figure 2:**
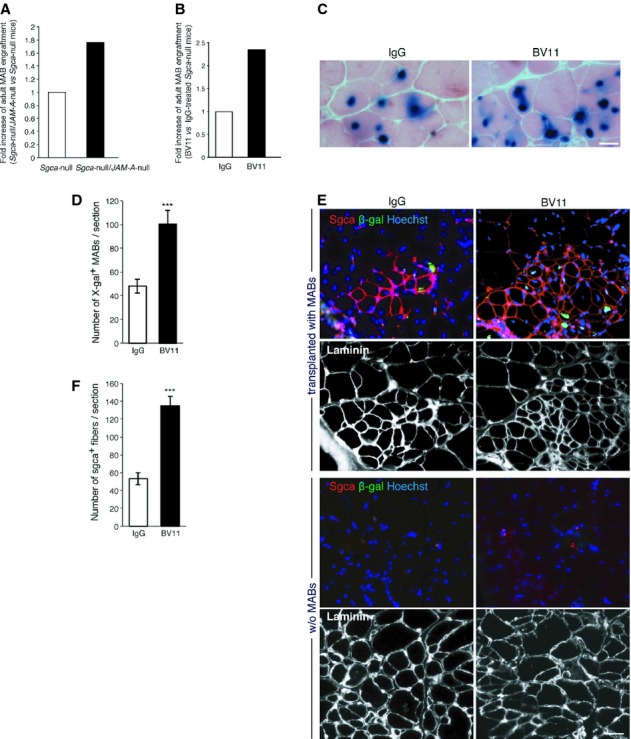
Adult MABs were injected into the right femoral artery of *Sgca*-null and *Sgca*-null/ *JAM-A*-null mice. After 6 h, the hind limb muscles (gastrocnemius, tibialis anterior and quadriceps) were collected and the presence of migrated cells was quantified using qRT-PCR with nLacZ primers. The nLacZ RNA relative level (normalized to GAPDH expression) obtained for controls was set to 1, and the ratios for *Sgca*-null/ *JAM-A*-null ( *n* = 8) *versus* control mice ( *Sgca*-null, *n* = 6) is shown. Fold increase has been extrapolated by data shown in Figure S1F.*Sgca*-null mice were treated with a JAM-A neutralizing antibody (BV11, 3 mg/kg) or with non-related IgG (IgG, 3 mg/kg) as control. After 1 h, the mice were intra-arterially transplanted with adult MABs and 6 h later the muscles were collected and processed as described in A. nLacZ RNA relative levels obtained for the controls was set to 1, and the ratio for BV11 *versus* control (IgG) is shown. Fold increase has been extrapolated by data shown in Figure S1G.BV11 ( *n* = 3) or IgG ( *n* = 3) were given to *Sgca*-null/scid/beige mice as described in B. Three weeks after the C57-nLacZ intra-arterial transplantations, the muscles were analyzed and representative cryosections of the gastrocnemius muscle stained for eosin and X-gal are shown. Scale bar: 40 μm.Quantification of the number of X-gal^+^ MABs in gastrocnemius cryosections. Scale bar: 40 μm. *** *P *< **0.001.Immunofluorescence staining of Sgca (red), β-galactosidase (β-gal, green), Hoechst (blue) and laminin (white, lower images of both panels) were performed on sections adjacent to those shown in C (transplanted with MABs) and on contralateral not transplanted muscles (w/o MABs) as a control. Merged images of red, green and blue signals are shown (upper images of both panels). Scale bar: 80 μm.Quantification of Sgca^+^ fibers shown in E, upper panel (transplanted with MABs). *** *P *< **0.0001. Data are expressed as means ± s.e.m.

The *in vivo* migration of MABs to the muscle tissue was then assessed in these *Sgca*-null and *Sgca*-null/ *JAM-A*-null mice, using adult murine MABs. Indeed, intra-arterially (femoral artery) transplanted MABs migrated significantly more efficiently to the muscles of the *Sgca*-null/ *JAM-A-*null mice than to those of the *Sgca*-null control mice (Fig 2A and supplementary Fig S1F).

Since JAM-A has been implicated in leukocyte extravasation, we investigated whether the absence of JAM-A modifies the inflammatory reaction of the dystrophic skeletal muscle. In contrast to MABs, the inflammatory infiltrate in skeletal muscle, which was mainly composed of CD68^+^ monocytes/macrophages, was reduced by 45% in the *Sgca-*null/ *JAM-A*-null mice compared to the *Sgca*-null mice (supplementary Fig S3C and D). This data confirms previous results that show how abrogation of both *JAM-A* expression and activity inhibits leukocyte infiltration in inflamed tissues (Corada *et al*, 2005). However to test whether the reduced number of macrophages could improve the engraftment of MABs indirectly by increasing cell survival and reduce muscle damage, we performed TUNEL staining. We found that apoptosis is not changed in *Sgca*-null/ *JAM-A-*null mice compared to *Sgca*-null mice (supplementary Fig S3E and F) suggesting that increased engraftment is not due to reduced cell death.

Finally, to develop a feasible future therapeutic approach, we investigated the acute inhibition of JAM-A function. *Sgca*-null dystrophic mice were treated with the BV11 anti-JAM-A neutralizing antibody (Martin-Padura *et al*, 1998; Del Maschio *et al*, 1999) or with non-related IgG, and after 1 h MABs were transplanted intra-arterially. Six hours after the transplantation, the efficiency of the engraftment was comparable to that measured in the *Sgca*-null/ *JAM-A*-null double mutants, with more than a doubling of the MAB engraftment in the mice treated with the blocking antibody (Fig 2B and supplementary Fig S1G). To rule out any possible adverse effects due to the acute administration of BV11 on vascular permeability *in vivo*, we looked for a vascular leakage in different organs (brain, liver and lung) as well as in skeletal muscle tissue of the mice treated with the blocking antibody. As a marker of increased permeability and edema we used fluorescent cadaverine (Maddaluno *et al*, 2013); we did not detect any significant accumulation of this tracer in the organs after BV11 treatment, including muscles, lungs, liver and brain (supplementary Fig S4A).

Overall, these data suggested an active contribution of the endothelium in MAB localization and prompted us to hypothesize that inhibition of JAM-A could enhance stem cell engraftment and thus muscle regeneration upon cell transplantation in muscular distrophies. To investigate this hypothesis, we performed intra-arterial transplantation of nLacz-C57 MABs in *Sgca*-null/scid/beige mice, an immunodeficient dystrophic model for LMGD2D that allows long-term engraftment of cells that will otherwise be rejected from the mouse host (e.g., human or GFP/β-galactosidase-expressing cells) (Tedesco *et al*, 2012). Specifically, the *Sgca*-null/scid/beige mice were pre-treated with BV11 or IgG and then transplanted with MABs. After 3 weeks, the mice were sacrificed and their hind limb muscles were collected. X-gal, β-galactosidase and Sgca staining were performed on gastrocnemius muscle sections, which confirmed that acute inhibition of JAM-A results in significantly improved engraftment of donor cells (Fig 2C and D) that subsequently differentiated in skeletal myofibers (Fig 2E upper panel and F; 1.4-fold increase). Of note, the presence of Sgca-positive fibers containing β-galactosidase-positive nuclei unequivocally demonstrated the contribution of the donor cells in generating the new skeletal myofibers. Moreover, *Sgca*-null/scid/beige mice do not display any revertant fibers (Fig 2E, lower panel w/o MABs), further confirming the donor origin of the Sgca-positive muscle fibers.

Altogether these results show that inhibition of JAM-A leads not only to an enhanced MAB engraftment, but as a consequence also to an increased formation of myofibers in a severe model of muscular dystrophy.

### Impairment of JAM-A increases murine and human MAB transmigration *in vitro*

To unravel the molecular mechanisms underlining the increased migration of MABs in the absence of JAM-A, we used an *in vitro* assay of MAB migration through cultured endothelial cells.

**Figure 3:**
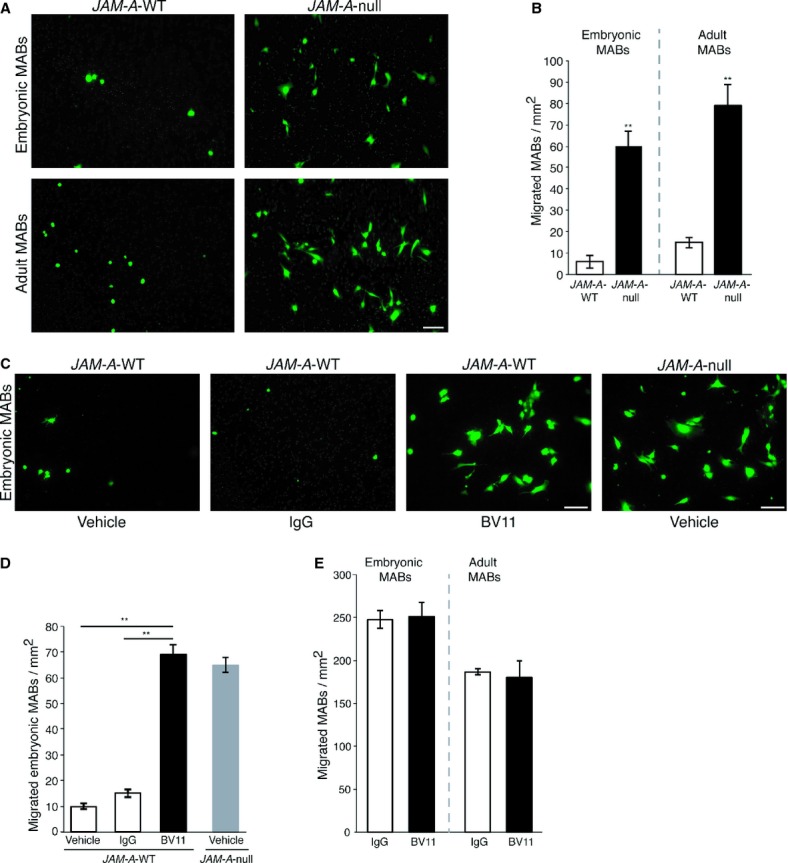
Endothelial cells isolated from *JAM-A*-WT (left) and *JAM-A*-null (right) mice were seeded onto glutaraldehyde-crosslinked gelatin-coated filters (Transwell). 6-carboxyfluorescin diacetate (6-CFDA)-labeled embryonic (top) and adult (bottom) MABs were added to the upper chamber and allowed to migrate for 6 h. The migrated MABs (green) on the lower sides of the filters were fixed and counted. A representative experiment of five independent experiments, each in triplicate, is shown. Scale bar: 100 μm.Quantification of the number of migrated MABs per area, as illustrated in A. ** *P *< **0.001. Data are means ± s.e.m. from five independent experiments, each in triplicate.As in A, except that *JAM-A*-WT endothelial cells were pre-treated (3 h) and further incubated (6 h) with vehicle, non-related IgG and BV11 (20 μg/ml) (left and middle, respectively). *JAM-A*-null endothelial cells were also incubated with vehicle (right) as a further internal control, illustrating that increased MAB transmigration due to acute inhibition of JAM-A is comparable with their capacity to migrate in the absence of JAM-A. Representative experiment of four independent experiments carried out in triplicate. Scale bars: 100 μm.Quantification of migrated embryonic MABs through *JAM-A*-WT (white and black bars) and *JAM-A*-null (grey bar) endothelial cells per area, as illustrated in C. ** *P *< **0.001. Data are means ± s.e.m. from four independent experiments, each in triplicate.6-CFDA-labelled embryonic (left) and adult (right) MABs were incubated with non-related IgGs (white bars), BV11 (20 μg/ml), (black bars) for 3 h. Then the MABs were seeded on filters and allowed to migrate for a further 3 h in the presence of the indicated agents. The migrated MABs on the lower side of the filters (green) were fixed and counted. Quantification of migrated cells per area is shown. Data are means ± s.e.m. from three independent experiments, each in triplicate.

We used endothelial cells isolated from the lungs of WT and *JAM-A*-null mice (Cera *et al*, 2004). These cells were grown as confluent monolayers on glutaraldehyde-cross-linked gelatin-coated filters. We then tested embryonic and adult murine MAB transmigration through these endothelial monolayers. Similar to what observed *in vivo*, the number of MABs that crossed the endothelium was significantly increased in the absence of JAM-A (Fig 3A and B). Furthermore, pre-incubation of the murine endothelial cells with BV11 strongly enhanced the transmigration of embryonic murine MABs (Fig 3C, left panel), with a 6-7-fold increase compared to the number of MABs that crossed the endothelium in the presence of vehicle or non-related IgG (Fig 3D). A similar increase in MAB transmigration was also achieved using monolayers of *JAM-A*-null endothelial cells, suggesting that chronic and acute JAM-A inhibition results in similar effects (Fig 3C and D).

Embryonic murine MABs are deficient in JAM-A and adult MABs express barely detectable levels of JAM-A (supplementary Fig S2B, left panel) making unlikely that MAB-associated JAM-A may play a role in the motility of these cells. However, to rule out this possibility, embryonic and adult MABs were incubated with BV11 or non-related IgG and tested for migration through filters that lacked an endothelial cell coating. As a result, inhibition of JAM-A did not change MAB transmigration (Fig 3E).

**Figure 4:**
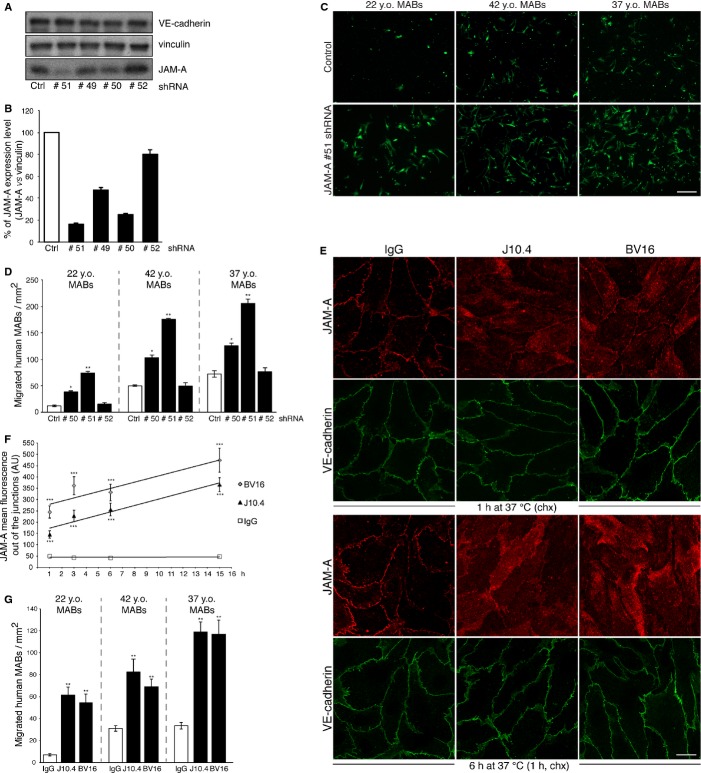
HUVECs with stable scrambled shRNA (Ctrl) or JAM-A targeting shRNAs (#51, #49, #50, #52) were generated (see Materials and Methods) and then homogenized. The cell lysates were analyzed by immunoblotting for JAM-A and VE-cadherin, using vinculin as loading control.Quantification of data presented in A. JAM-A expression levels were normalized with vinculin and are expressed as percentages. Data are means ± s.d. from three independent experiments.HUVECs with stable scrambled shRNA (ctrl) or a JAM-A targeting shRNA (#51) were seeded onto Transwell filters for 72 h. 6-CFDA-labeled human MABs derived from three different donors (22-, 42-and 37-year old [y.o.] healthy donors) were added to the upper chamber and allowed to migrate for 8 h. Migrated MABs on the lower sides of the filters (green) were fixed and counted. Representative data are shown from four independent experiments, each in triplicate. Scale bar: 100 μm.Quantification of migrated MABs per area is shown for 22 y.o. (left), 42 y.o. (middle) and 37 y.o. (right) MABs. * *P *< **0.01, ** *P *< **0.001. Data are means ± s.e.m. from four independent experiments, each in triplicate.Confluent monolayers of HUVECs were treated with non-related IgG, J10.4 and BV16 (12 μg/ml) at 37°C, for the indicated times. The cells were then incubated for the last 1 h with 100 μg/ml cycloheximide (chx) to inhibit protein synthesis, fixed and stained for JAM-A (red) and VE-cadherin (marker for junctions, green). Scale bar: 20 μm.Quantification of JAM-A delocalization. Time course (1, 3, 6 and 15 h) for mean fluorescence (expressed in arbitrary units, AU, in cells treated with IgG, J10.4 and BV16, as indicated). The trend lines are shown. *** *P *< **0.00001 compared with IgG. Data are means ± s.e.m. from three independent experiments.Experiments performed as in C, except the HUVECs were pre-treated (3 h) and further incubated (8 h) with non-related IgG, J10.4 and BV16 (as indicated). Quantification of migrated MABs per area is shown for 22 y.o. (left), 42 y.o. (middle) and 37 y.o. (right) MABs. ** *P *< **0.001. Data are means ± s.e.m. from three independent experiments, each in triplicate. Source data are available for this figure.

We then extended the study to human cells. For this, we first generated human umbilical vein endothelial cells (HUVECs) that were stably infected with lentiviral vectors expressing shRNA against *JAM-A*. Four different shRNA constructs were tested to establish stable *JAM-A* deficient cell lines. The efficiency of the different constructs was evaluated using Western blot (Fig 4A) and the relative densitometry showed that sh#50 and sh#51 RNAs significantly reduced JAM-A protein expression by approximately 75–85%, as compared to the control (Fig 4B). The sh#50, sh#51 and sh#52 RNAs were then selected to assess the impact of *JAM-A* down-regulation on human MAB transmigration. The human MABs were derived from three healthy donors and were selected for their different spontaneous myogenic differentiation into skeletal myosin heavy chain positive-myotubes (supplementary Fig S2C). Moreover, as we previously reported for murine MABs, Western blot analysis showed only a faint band corresponding to JAM-A in 37 years old (y.o.) human MABs, while 22 y.o. and 42 y.o. MABs did not express JAM-A (supplementary Fig S2B, right panel). Consistent with the data obtained with murine cells, the human MABs migrated more efficiently when the endothelial JAM-A was reduced and the increase in cell transmigration correlated with the efficiency of JAM-A depletion in HUVECs, suggesting a dose-dependent effect (Fig 4B-D).

To evaluate the potential use of blocking antibodies against human JAM-A, we then studied the biological activity of a commercially available human JAM-A-neutralizing antibody, J10.4 (Mandell *et al*, 2004), along with that of BV16 (produced in our laboratory) (Williams *et al*, 1999), in terms of their ability to dismantle JAM-A from endothelial junctions. As shown in Fig 4E, both antibodies altered JAM-A localization to a similar extent. One hour after J10.4 or BV16 treatment of endothelial cells, the JAM-A staining appeared to be more diffuse and less intense at the junctions. This phenomenon increased over time (Fig 4E and F). Furthermore, these JAM-A-neutralizing antibodies induced comparable strong increases in human MAB transmigration (Fig 4G).

Taken together, these results support the use of JAM-A blocking antibodies to improve murine and human MAB engraftment.

### Changes in junction organization drive MAB transmigration through *JAM-A*-null endothelial cells

Cell extravasation involves a cascade of events initiated by adhesion between flowing cells and the vascular endothelium and followed by cell diapedesis through the endothelial monolayer. To investigate which of these steps are modified during MAB transmigration through *JAM-A*-null endothelial cells, we first performed adhesion assays. As reported in Fig S5A, embryonic MABs adhere in a similar manner to the endothelium regardless of the presence or the absence of JAM-A, while adult MABs adhere even less to *JAM-A*-null endothelial cells. This strongly suggests that increased adhesion is not responsible for the greater extravasation of the cells. Then, we asked whether the absence of JAM-A could modulate β1-and β3-integrin expression or activity as previously reported in other systems (Naik & Naik, 2006; Severson *et al*, 2009; McSherry *et al*, 2011; Peddibhotla *et al*, 2013). Western blot analysis showed that the absence of JAM-A resulted in slightly decreased levels of β1-integrin, as previously reported (Severson *et al*, 2009; McSherry *et al*, 2011). In contrast, the expression level of β3 integrin was increased in *JAM-A*-null endothelial cells compared to control cells (supplementary Fig S5B and C). Changes in β1 and β3 integrin expression levels in the endothelium may induce a different distribution of extracellular matrix proteins in *JAM-A*-null and WT endothelial cells which, in turn, may modify MAB migration. To test this hypothesis, we analyzed the organization of three major members of the endothelial basement membrane: fibronectin, laminin and collagen IV but we could not observe significant differences comparing *JAM-A*-null and WT endothelial cells (supplementary Fig S5D). Moreover, MABs migrated in a comparable way across filters coated by the extracellular matrix of *JAM-A*-null and WT endothelial cells (see Materials and Methods and supplementary Fig S5E).

Finally, we found that MAB transmigration through *JAM-A-*null endothelial cells was not significantly affected by the presence of β1-or β3-integrin blocking antibodies, compared to non-related IgG (supplementary Fig S5F). Overall these results make unlikely the involvement of matrix or β1-or β3-integrins in the increased migration of MABs through *JAM-A*-null endothelial monolayers.

**Figure 5:**
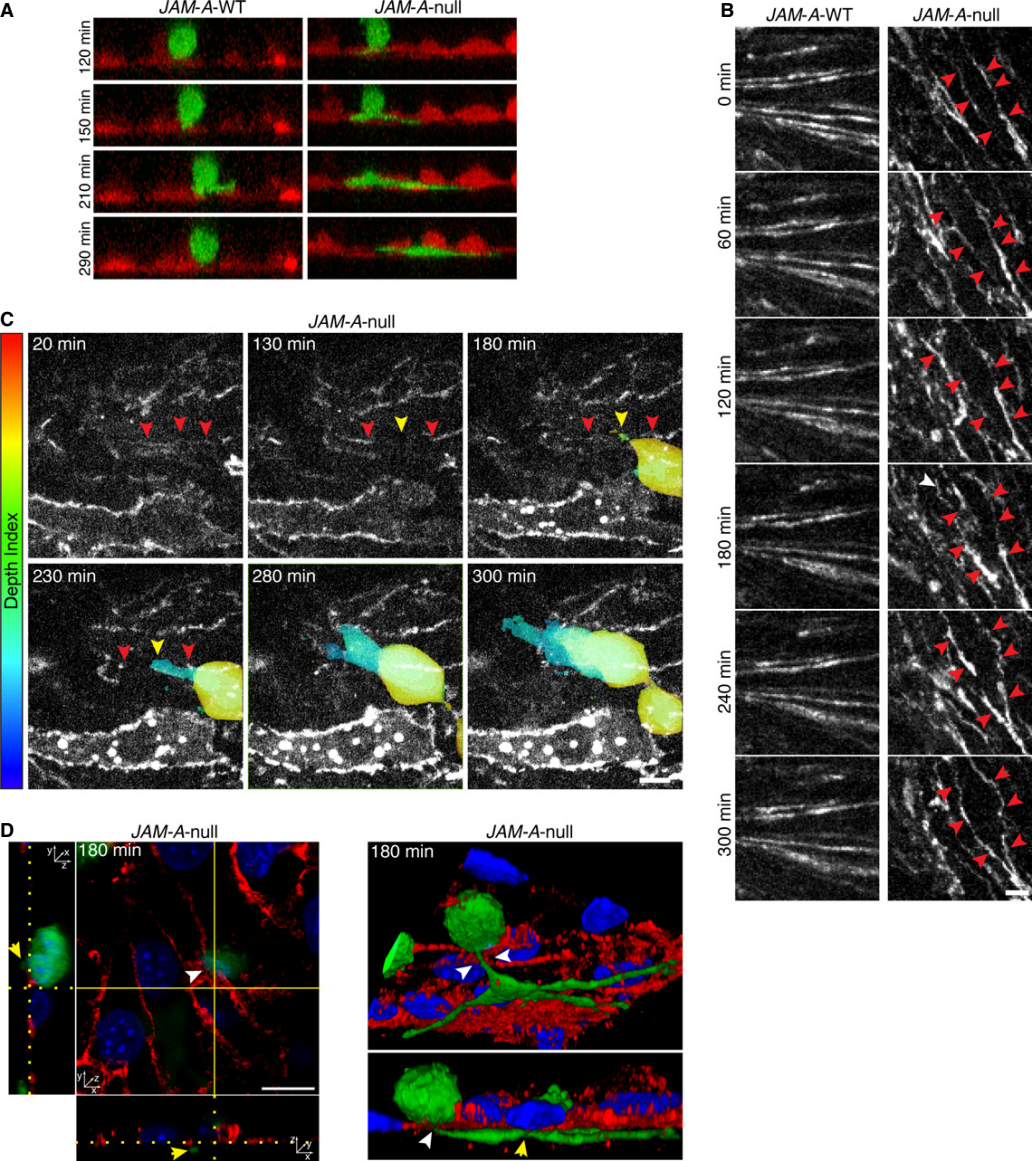
Time-lapse imaging of adult MAB (C57-GFP, green) transmigration across *JAM-A*-WT (left, red) and *JAM-A*-null (right, red) endothelial cells expressing Td-tomato seeded onto collagen matrix. Images stack were obtained every 7 min from 0 (time of MAB addition to the endothelial monolayers) to 380 min. Representative time frame images (120, 150, 210 and 290 min) from stack projection along *y*-axis are shown. See also supplementary Fig S5G and Movie S1.Time-lapse imaging of *JAM-A*-WT (left) and *JAM-A*-null (right) endothelial cells expressing PECAM-1-GFP as marker of cell-cell junction (white). Images stack were obtained every 12 min for 300 min. Representative time frame images (0, 60, 120, 180, 240 and 300 min) are shown. The red arrowheads indicated the junction dynamics. Scale bar: 10 μm. See also supplementary Movie S2.Time-lapse imaging of C57-GFP MAB transmigration across *JAM-A*-null endothelial cells expressing PECAM-1-GFP (white) seeded onto collagen matrix. Images stack were obtained every 10 min for 300 min. Representative time frame images (20, 130, 180, 230, 280, 300 min) are shown. MAB is shown as a gradient of pseudo-colors (Depth index) ranging from red (top, on the endothelium) to blue (bottom, through the collagen matrix), varying according to focal plane depth. The red arrowheads indicated the junctions. The yellow arrowhead highlights the crossing point of MAB through the disrupted junctions. Scale bar: 10 μm. See also supplementary Movie S3.Time course of C57-GFP MAB transmigration across *JAM-A*-null endothelial cells seeded onto collagen matrix. *JAM-A*-null endothelial cells were fixed at different time point during the transmigration assay and then stained with anti-PECAM-1 (red) as marker of cell-cell junction and DAPI (blue) as marker of nuclei. Two representations of a confocal *z*-stack taken after 180 min of MAB transmigration are shown. On the left, orthogonal cross-sections and on the right, a couple of images taken from a 3D reconstruction are shown. The white arrowhead (top) indicated the crossing point of MABs through the disorganized junctions. The yellow arrowheads highlighted the portion of MAB under the endothelium. Scale bar: 10 μm.

To further understand the dynamics of MAB transmigration through endothelial cells we followed the time course of this process in live-cell imaging. Both *JAM-A*-WT and *JAM-A*-null endothelial cells were stably transfected with the fluorescent protein Td-Tomato to facilitate the observation of GFP transduced MABs crossing the endothelial monolayers and invading the matrix *in vitro*. We found that MABs make contact with the *JAM-A*-WT endothelialium but they are unable to cross the monolayer and retain a spherical shape up to 290 min. In contrast, MABs seeded on *JAM-A*-null endothelium are able to elongate pseudopods through the endothelial monolayer, transmigrate and invade the collagen gel (Fig 5A, supplementary Fig S5G and Movie S1).

We then investigated whether the absence of JAM-A may change the organization of endothelial junctions. To this end we performed live-cell imaging of both *JAM-A*-WT and *JAM-A*-null endothelial cells stably transfected with AcGFP-tagged PECAM-1. During 5 h of observation, we found that junctions in *JAM-A*-null endothelial monolayers are continuously formed and disrupted (Fig 5B and supplementary Movie S2) showing a more dynamic behavior compared to *JAM-A*-WT endothelium. These observations suggest that the continuous remodeling of junctions in *JAM-A*-null endothelial cells allows MABs transmigration through them. Evidence of MABs crossing endothelial cell junctions in the region where they are disassembled is reported in Fig 5C and supplementary Movie S3.

Finally, to further confirm that MABs crossed the endothelial monolayers through the junctions, we performed a time course transmigration assay of C57-GFP MABs through *JAM-A*-null endothelial cells. The junctions were stained with an anti-PECAM-1 antibody. The orthogonal view and the 3 Dimentional reconstruction showed that C57-GFP MABs transmigrate across the endothelium via the paracellular route (Fig 5D) mediated by local disruption of junctions.

Taken together these data support the idea that MABs transmigration occurs through endothelial junctions and that this process is significantly increased when JAM-A is absent and junctions become less tightly organized.

### JAM-A regulates the small GTPase Rap-1 in endothelial cells

**Figure 6:**
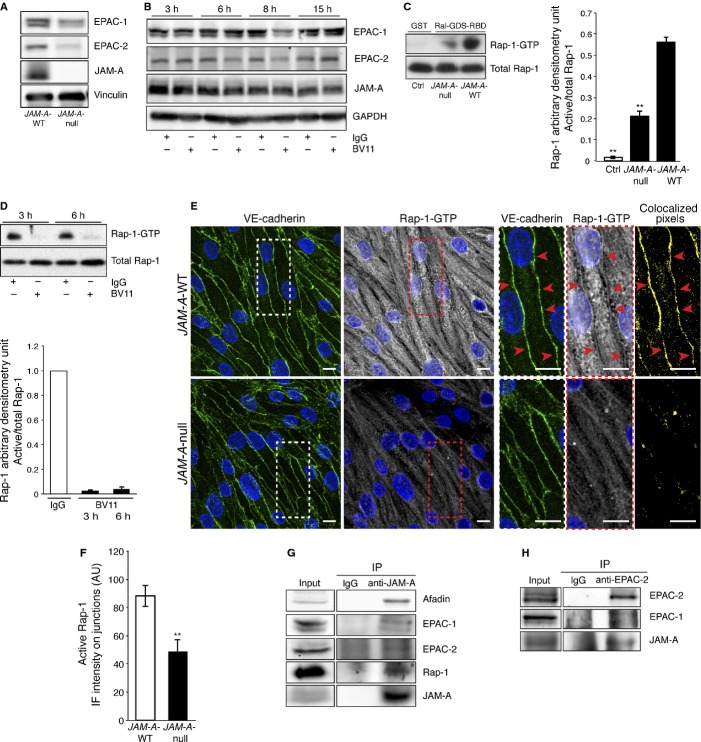
Cultured endothelial cells isolated from *JAM-A*-WT and *JAM-A*-null mice were homogenized. The lysates were analyzed by immunoblotting for EPAC-1, EPAC-2 and JAM-A using vinculin as a loading control.*JAM-A*-WT endothelial cells were incubated with IgG or BV11 (20 μg/ml) for the indicated times and then treated as in A, except we used GAPDH as the loading control. Representative experiment of three independent experiments is shown.Confluent monolayers of *JAM-A*-WT and *JAM-A*-null endothelial cells were harvested in lysis buffer. The protein extracts were incubated with the Ral-GDS-RBD probe or the GST-negative control (as indicated). The active Rap-1 (Rap-1-GTP) and total Rap-1 were detected using an anti-Rap-1 antibody. Representative data are shown for the densitometry analysis of active Rap-1-GTP, normalized for total protein. ** *P *< **0.001 versus *JAM-A*-WT. Data are means ± s.d. from three independent experiments.*JAM-A*-WT endothelial cells were incubated with IgG or BV11 for 3 h and 6 h (as indicated). Rap1-GTP loading quantified by GST pull-down/ Western blotting and densitometry analysis, as reported in C. Data are means ± s.d. from three independent experiments.Confluent monolayers of *JAM-A*-WT and *JAM-A*-null endothelial cells were fixed and double-stained with anti-VE-cadherin (green, left) as marker of cell-cell junction and GST-RalGDS-RBD (white) to detect the Rap-1-GTP (right). The insets show high magnification fields of VE-cadherin (white, dashed square) and Rap-1-GTP (red, dashed square) stainings. The arrowheads indicated the junctions. Pixels representing the co-localization of Rap-1-GTP with VE-cadherin are highlighted in yellow. Scale bar: 20 μm.Rap-1-GTP immunofluorescence intensity at junctions is expressed as arbitrary units (AU). ** *P *< **0.02. Data are means ± s.e.m. from three independent experiments.Cultured *JAM-A*-WT endothelial cells were homogenized, and the lysates (Input) were subjected to immunoprecipitation (IP) using an anti-JAM-A monoclonal antibody or IgG, as control. Precipitated material was analyzed by immunoblotting for JAM-A, afadin, EPAC-1, EPAC-2 and Rap-1. Representative experiment of three independent experiments is shown.As in G, except the lysates (Input) were subjected to immunoprecipitation (IP) using an anti-EPAC-2 antibody or IgG, as control. Precipitated material was analyzed by immunoblotting for JAM-A, EPAC-1 and EPAC-2. Representative experiment of three independent experiments is shown. Source data are available for this figure.

To uncover the JAM-A transduction machinery, we first sought to identify transcripts regulated by JAM-A expression. For this, we carried out Affymetrix gene expression analysis to compare endothelial cells isolated from lungs of WT and *JAM-A*-null mice. Among the prominently affected genes, cAMP-responsive Rap-1 guanine nucleotide exchange factors (GEFs), such us EPAC-1 and EPAC-2, showed significant down-regulation in the absence of JAM-A (local-pooled-error test, *P *< **0.05; fold-change >2). The differences in the expression levels were confirmed at the protein level by immunoblotting (Fig 6A), and down-regulation of EPAC expression was observed upon inhibition of JAM-A with the BV11 blocking antibody (Fig 6B).

The down-regulation of EPAC-1 and EPAC-2 raised the possibility that JAM-A inhibition might affect the activation of the small GTPase Rap-1 in endothelial cells, as previously shown in epithelial cells (Mandell *et al*, 2005; Severson *et al*, 2009; McSherry *et al*, 2011). To address this issue, we first examined the activation of Rap-1 in endothelial cells derived from *JAM-A*-null mice using a pull-down assay for active GTP-loaded Rap-1 (Rap-1-GTP). To detect Rap-1-GTP we used a glutathione *S*-transferase (GST) fusion protein containing the RalGDS-Rap-1-binding domain (RalGDS-RBD). The bound protein was analysed by Western blotting, which showed that Rap-1-GTP levels decreased by 60% in the *JAM-A*-null endothelial cells, as compared to the *JAM-A*-WT cells, whereas the total level of Rap-1 was unaffected (Fig 6C). In a subsequent experiment, endothelial cells were treated with BV11 or with non-related IgG as control. The Western blotting showed a reduction in active Rap-1, 3 h after BV11 administration, and this effect lasted for up to 6 h following JAM-A inhibition (Fig 6D). To investigate whether JAM-A regulates Rap-1 localization to specific cell compartments, we examined the localization of Rap-1-GTP by confocal microscopy using GST-RalGDS-RBD as a probe in combination with an anti-GST antibody for specifically detecting the Rap-1-GTP. Consistent with the results of the pull-down assay, the absence of JAM-A reduced the activation of Rap-1 both in the perinuclear regions and at cell-cell contacts (defined by VE-cadherin staining; Fig 6E and F). These data indicate that JAM-A regulates not only the activation, but also the junctional localization of active Rap-1 in endothelial cells.

Using immunoprecipitation analysis, we showed that JAM-A is associated to EPAC-1, EPAC-2, Rap-1 and afadin (a JAM-A-interacting protein) (Bazzoni *et al*, 2000; Ebnet *et al*, 2000; Severson *et al*, 2009), which suggested that these proteins belong to a JAM-A multiprotein complex that controls the organization of junctions (Fig 6G and H). However, others have not been able to detect any co-association between JAM-A and Rap-1 (Severson *et al*, 2009; McSherry *et al*, 2011), which appears to be due to differences in the protocols used for immunoprecipitation, potentially in terms of the use of cross-linking or lower levels of starting materials (see Materials and Methods).

These data suggest that JAM-A promotes the activation of Rap-1 by tethering EPAC-1 and EPAC-2.

### Rap-1 modulation controls MAB migration through the endothelium *in vitro* and improves muscular functionality *in vivo*

**Figure 7:**
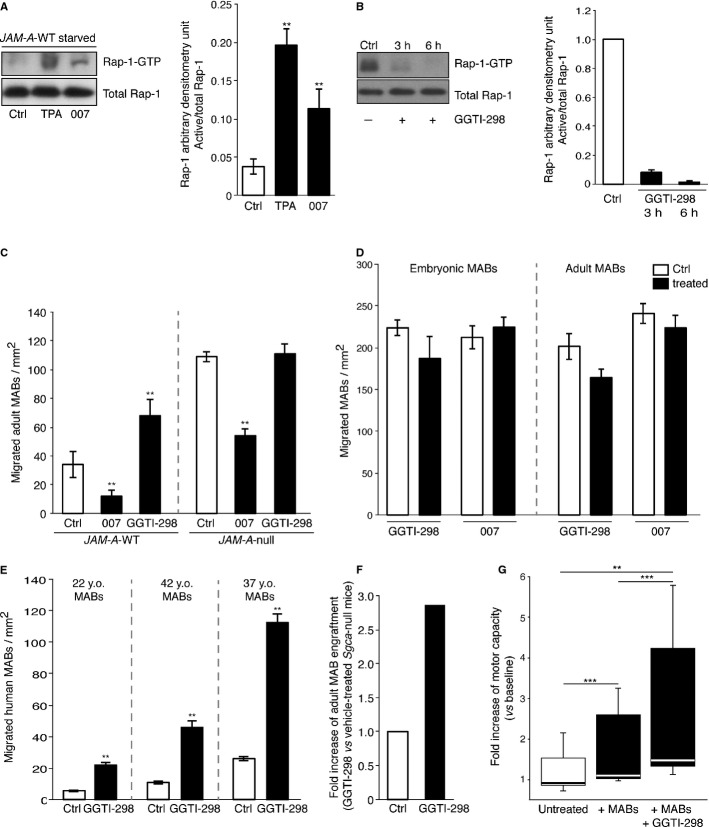
*JAM-A*-WT endothelial cells were starved for 16 h and then treated with vehicle (ctrl), a selective activator of EPAC (007, 100 μM) or tetradecanoylphorbol acetate (TPA, 10 μM), an activator of the signal transduction enzyme protein kinase C, used as positive control for Rap-1 activation. Rap1-GTP loading was quantified by GST pull-down/ Western blotting and densitometry analysis. Representative blot (left) and quantification of densitometry analysis (right) of Rap-1-GTP levels normalized for total amount of protein. ** *P *< **0.001. Data are means ± s.d. from three independent experiments.*JAM-A*-WT endothelial cells were incubated with the Rap-1 pharmacological inhibitor GGTI-298 (10 μM) or vehicle for 3 h and 6 h (as indicated). Rap1-GTP loading was quantified as in A. Data are means ± s.d. from three independent experiments. Note: to highlight Rap-1 activation, the filter in A was exposed for 30 s, while in B it was exposed for 5 min, to improve the evaluation of Rap-1 inhibition at 3 h and 6 h.*JAM-A*-WT and *JAM-A*-null endothelial cells seeded on coated filters for 72 h and treated with vehicle (ctrl) or 007 or GGTI-298 for the last 30 min, as indicated. 6-CFDA-labelled adult MABs were added to the upper chamber and allowed to migrate for 6 h under these conditions. Quantification of migrated MABs per area for *JAM-A*-WT (on the left of the dashed line) and *JAM-A*-null (on the right of the dashed line) endothelial cells. ** *P *< **0.001. Data are means ± s.e.m. from three independent experiments, each in triplicate.6-CFDA-labelled embryonic (left) and adult (right) MABs were incubated with vehicle (ctrl, white bars), 007 (100 μM) or GGTI-298 (10 μM) (black bars) for 3 h, as indicated. Then the MABs were seeded on filters and allowed to migrate for a further 3 h in the presence of the indicated agents. The migrated MABs on the lower side of the filters (green) were fixed and counted. Quantification of migrated cells per area is shown. Data are means ± s.e.m. from three independent experiments, each in triplicate.HUVECs were treated as described in C. Quantification of migrated MABs per area is shown for 22 y.o. (left), 42 y.o. (middle) and 37 y.o. (right) MABs. ** *P *< **0.001. Data are means ± s.e.m. from three independent experiments, each in triplicate.*Sgca*-null mice were treated with GGTI-298 (50 mg/Kg, *n* = 2) or with vehicle (ctrl, *n* = 3) for 1 h and then were intra-arterially transplanted with adult MABs. After 6 h, the hind limb muscles were collected and the presence of migrated cells was quantified using qRT-PCR with nLacZ primers. The relative RNA level of nLacZ obtained for control was set to 1, and the ratio for GGTI-298 versus control is shown. Fold increases have been extrapolated by data shown in Figure S1H.Box-plot showing exercise tolerance in a treadmill test for untreated *Sgca*-null/scid/beige mice, *Sgca*-null/scid/beige mice transplanted with MABs (+ MABs, 5 × 10^5^ cells injected bilaterally in femoral arteries) and *Sgca*-null/scid/beige mice transplanted with MABs and treated with GGTI-298 (+MABs + GGTI-298, 5 × 10^5^ cells injected bilaterally in femoral arteries). Values are plotted as fold increase of motor capacity measured at different time points (21, 28 and 35 days post transplantation), and were normalized with the baseline performances of each mouse. Data are means ± s.e.m. of 3 mice per group. ** *P *< **0.01, *** *P *< **0.001, one-way ANOVA. Source data are available for this figure.

As the data above show that JAM-A acts by stimulating Rap-1 activity, we asked whether also the trans-endothelial migration of MABs could be modulated by Rap-1. To address this question, we used the selective activator of EPAC, 007, and the Rap-1 pharmacological inhibitor GGTI-298. As shown in Fig 7A, Rap-1-GTP increased in the presence of 007, as also seen for tetradecanoylphorbol acetate (TPA), an activator of the signal transduction enzyme protein kinase C used here as a positive control. On the other hand, Rap-1 activation was strongly inhibited in the presence of GGTI-298 (Fig 7B). Following Rap-1 activation via 007, the transmigration of adult murine MABs was significantly reduced by some 60% (Fig 7C). Conversely, the transmigration of MABs was significantly doubled in the presence of the Rap-1 inhibitor GGTI-298, which suggested an active contribution of Rap-1 in MAB extravasation. Interestingly, incubation of the *JAM-A*-null endothelial cells with 007 significantly counteracted the ability of MABs to migrate through the endothelium (50% inhibition), which supports the concept that Rap-1 might by-pass JAM-A in inhibiting MAB transmigration (Fig 7C). Conversely, GGTI-298 treatment had no effect on *JAM-A*-null endothelial cells (Fig 7C).

Overall these data are consistent with the idea that Rap-1 is the downstream effector of JAM-A in the modulation of MAB transmigration through endothelial junctions. Furthermore, MABs pre-incubated with 007 or GGTI-298 or vehicle showed no significant differences in their transmigration through filters lacking endothelial cells, excluding the possibility that these agents have a direct effect on MAB migration (Fig 7D). Importantly, GGTI-298 was also effective in an *in vitro* human setting, since it enhanced transmigration of human MABs through human endothelium (Fig 7E).

The data reported above support the idea that inhibition of Rap-1 may improve MAB engraftment. To validate these data *in vivo*, we first performed a Rap-1 pull-down assay after intra-peritoneal administration of GGTI-298 and we found that Rap-1 activity was inhibited in skeletal muscle of *Sgca-*null mice (supplementary Fig S4B) as expected. GGTI-298 inhibited Rap-1 activation to a similar extent as BV11 (supplementary Fig S4B). We also observed that GGTI-298 did not induce tissue edema (supplementary Fig S4A).

We then administrated GGTI-298 to *Sgca-*null mice before MAB transplantation and we found that MABs engrafted more efficiently upon this treatment, confirming *in vitro* data (Fig 7F and supplementary Fig S1H).

Most importantly, functional experiments performed on dystrophic, immunodeficient age-matched (2 months old; 4 months old; 7 months old) *Sgca*-null/scid/beige mice showed that treating them with GGTI-298 prior to MABs intra-arterial injection significantly improved exercise tolerance. Indeed treated animals (GGTI-298 + MABs) showed enhanced motor capacity, running from 33 to 72% more than control animals (2.5 fold increase versus baseline, ** *P *< **0.01) and from 16 to 55% more than treated animals (+ MABs) (1.5 fold increase versus baseline, *** *P *< **0.001) (Fig 7G and supplementary Fig S6).

Altogether, these data provide evidence that JAM-A and its downstream effector Rap-1 are important biological targets for improving MAB engraftment in dystrophic muscles. These observations open the possibility of using inhibitors of JAM-A or Rap-1 to improve the efficacy of stem cell therapy in tissue regeneration.

## Discussion

The efficacy of both autologous and allogeneic stem cell therapy for tissue regeneration is linked to the amount of cells that engraft into the damaged area. Here we introduce a novel strategy to increase this function by targeting endothelial cell-to-cell junctions. These structures have crucial roles in the regulation of leukocyte diapedesis through the vessel wall (reviewed in Nourshargh *et al*, 2010). Several proteins form the architecture of intercellular junctions in the endothelium, and among these, as shown in the present study, the tight junction adhesive protein JAM-A markedly limits MAB extravasation (reviewed in Weber *et al*, 2007).

Targeting *JAM-A* expression or function strongly increases MAB transmigration through endothelial cells and their engraftment into dystrophic muscle. This results in a greater contribution of the donor cells to the regeneration of the host muscle.

The specificity of the action of JAM-A in limiting the passage of MABs is supported by different sets of data. First, inactivation of PECAM-1, another junctional protein known to increase leukocyte extravasation, was not as effective as JAM-A in increasing MAB engraftment. Moreover, inhibitors of the specific downstream signaling pathway of JAM-A had the same effect obtained by inactivating the protein itself.

JAM-A is known to act by inducing and maintaining endothelial cell junction organization (Ebnet *et al*, 2004; Nourshargh *et al*, 2010). Early studies showed that JAM-A is the first junctional component to concentrate at intercellular junctions when endothelial cells come into contact (Bazzoni & Dejana, 2004; Iden *et al*, 2012). Therefore it is likely that through specific intracellular signaling JAM-A promotes a correct organization of junctions. This concept is supported by the data reported here showing a more dynamic and less tight organization of endothelial junctions in absence of JAM-A. Consistently, MABs are able to cross endothelial cells through junctions and this process is much more efficient in absence of JAM-A. Additionally, other JAM-A functions such as endothelial cell adhesion through integrins (Naik & Naik, 2006; Severson *et al*, 2009; McSherry *et al*, 2011; Peddibhotla *et al*, 2013) or changes in matrix composition do not appear to play a major role in modulating MAB extravasation. Therefore, our data support the concept that MABs extravasate more efficiently in absence of JAM-A since endothelial cell junctions are less tight.

Through the analysis of the gene expression profiles, we initially showed that in the absence of JAM-A, endothelial cells express lower levels of EPAC-1 and EPAC-2. These proteins are potent activators of the small GTPase Rap-1, which maintains the correct organization of cell-cell junctions and increases endothelial barrier function (Cullere *et al*, 2005; Kooistra *et al*, 2007; Dube *et al*, 2008). In the absence of JAM-A, Rap-1 activation and junctional localization are inhibited. As a consequence, junctions are partially dismantled and MAB extravasation is increased. Comparable results were obtained by inhibiting Rap-1 directly. These data are in agreement with a previous study that showed a link between JAM-A expression and Rap-1 activation in other systems (Mandell *et al*, 2005; Severson *et al*, 2009; McSherry *et al*, 2011) although the mechanism of action of JAM-A in Rap-1 activation remained unsolved (Monteiro & Parkos, 2012).

Here we show that JAM-A acts by sustaining the expression of EPAC-1 and EPAC-2, which are in turn Rap-1 activators. This effect can be inhibited not only by abrogating *JAM-A* expression, as in the *JAM-A*-null cells or mice, but also by promoting the release of JAM-A from endothelial junctions through incubations with blocking antibodies. This last observation suggests that for sustained *Epac* gene expression, JAM-A needs to be present and functionally active at the endothelial junctions.

The data reported here describe a novel strategy of intervention to increase MAB engraftment in dystrophic muscle. Chemical inhibitors of Rap-1 and JAM-A-blocking antibodies can be administered before MAB transplantation to improve the MAB infiltration into the damaged muscle. To this end, we extended our studies to a human system by testing chemical inhibitors of Rap-1 and JAM-A-blocking antibodies on the transmigration of human MABs through human endothelial cell monolayers. We show similar results comparing the murine and human systems, supporting the possibility of translating these findings to a clinical setting. In this context the availability of clinically approved antibodies or drugs could speed up further translational studies. This would be of great relevance, as increased engraftment strictly correlates with increased number of newly generated skeletal myofibers. Indeed, functional experiments performed on dystrophic, immunodeficient *Sgca*-null/scid/beige show that treating mice with the Rap-1 inhibitor significantly improved their motor capacity and exercise tolerance.

Thus, improved MAB localization into the damaged muscle will promote further the clinical efficacy of these treatments. This must now await the availability and use of clinically approved antibodies or drugs.

It was shown that JAM-A actively participates in leukocyte diapedesis through endothelial junctions. Consistent with other reports (Corada *et al*, 2005; Woodfin *et al*, 2007), we could confirm in the present studies that when JAM-A is inhibited, fewer monocytes can infiltrate into the inflamed muscle. It is not surprising that mechanisms that regulate cell adhesion and extravasation may vary in different cell types. A possible explanation for the opposite behavior of MABs and leukocytes is that leukocytes express JAM-A and the homotypic interaction with endothelial JAM-A was found to be important in directing their diapedesis through endothelial junctions (Weber *et al*, 2007; Nourshargh *et al*, 2010; Vestweber, 2012a,2012b). In contrast, MABs do not express JAM-A and cannot interact with it on the endothelial surface.

In conclusion, we have unveiled a mechanism through which donor MAB engraftment can be modulated by acting on host endothelium. While the focus of our study is on MAB engraftment in muscular dystrophy, the strategy that we have described might also have a more general impact. For instance, in preliminary studies, we have seen that JAM-A inhibition can markedly increase MAB infiltration in the ischemic heart and improve the recovery of this organ. Furthermore, we cannot exclude that JAM-A inhibition will increase transmigration and engraftment of other types of stem/ progenitor cells, thus improving cell-based therapies of further, different diseases.

## Materials and Methods

### Mice

*PECAM-1*-null (Duncan *et al*, 1999), *JAM-A*-null (Cera *et al*, 2004; Corada *et al*, 2005), *Sgca*-null (Duclos *et al*, 1998) and *Sgca*-null/scid/beige (Tedesco *et al*, 2012) mice (age-mached within each experiment) were used for these studies. *Sgca*-null/ *JAM-A*-null transgenic mice were generated by crossing *Sgca*-null and *JAM-A*-null mice, and by selecting homozygous mice for both genes. All of the mice used in this study were on a C57BL/6J background, except for the *Sgca*-null/scid/beige mice, and were genotyped to verify the mutations. Genotyping PCR for *Sgca* (WT allele 1066 bp; mutated allele 618 bp) and *JAM-A* (WT allele 800 bp; mutated allele 500 bp) mutations were detected with the primers listed in supplementary Table S1.

For *Sgca*-null/scid/beige and *PECAM-1*-null mice genotyping see Tedesco *et al* (2012) and Duncan *et al* (1999), respectively. All of the procedures involving living animals conformed to Italian (D.L.vo 116/92 and subsequent additions) and English law (Animals Scientific Procedure Act 1986 and subsequent additions) and were approved respectively by the San Raffaele Institutional Review Board (IACUC 355) and UK Home Office (Project License PPL no. 70/7435). C57BL/6J WT mice were purchased from Charles River Laboratories.

### Cell culture

Endothelial cells were isolated from lungs of *JAM-A*-WT and *JAM-A*-null mice, cultured and immortalized as described previously (Cera *et al*, 2004). Murine endothelial cells were treated with the JAM-A-neutralizing antibody BV11 (20 μg/ml), β1-integrin (10 μg/ml), β3-integrin (3 μg/ml) blocking antibodies, non-related IgG (20 μg/ml, 10 μg/ml or 3 μg/ml) for the established time in Dulbecco's modified Eagle's medium (DMEM) supplemented with 2% fetal bovine serum (FBS) and then used in the assays. Cell monolayers were starved in DMEM containing 0.5% serum for 16 h prior to treatments with an activator of the signal transduction enzyme protein kinase C tetradecanoylphorbol acetate (TPA, 10 μM) (Sigma-Aldrich, St Louis, MO, USA) for 10 min, EPAC activator (8-pCPT-2′-O-Me-cAMP, 100 μM, referred to as ‘007’ throughout the manuscript, in keeping with other publications and for simplicity) (Biolog, Bremen, Germany) for 5 min, and Rap-1 inhibitor (GGTI-298, 10 μM dissolved in dimethyl sulfoxide containing 10 mM dithiothreitol, as suggested by the supplier) (Calbiochem, Merck Millipore, Darmstadt, Germany) for 1 h, and then used in the assays.

HUVECs were isolated from umbilical veins and cultured as previously described (Grazia Lampugnani *et al*, 2003). Human endothelial cells were treated with the JAM-A-neutralizing antibodies J10.4 (12 μg/ml) and BV16 (12 μg/ml), or with non-related mouse IgG (12 μg/ml), for 3 h in MCDB-131 medium supplemented with 10% FBS, and then used for the *in vitro* migration assays. In immunofluorescence experiments, the HUVECs were incubated with cycloheximide (100 μg/ml) (Sigma-Aldrich), to inhibit protein synthesis, for the last 1 h, before fixation. Cell monolayers were starved in MCDB-131 containing 0.5% serum for 3 h prior to treatments with GGTI-298 (10 μM) for 1 h, and then used for the *in vitro* migration assays. All of the treatments were maintained during the assays.

Both murine (embryonic D16-GFP and adult C57-nLacZ or C57-GFP) and human MABs (derived respectively from 22-, 42-and 37-years old healthy donors) were cultured as previously reported (Tonlorenzi *et al*, 2007). Detailed protocols fo *r in vitro* skeletal muscle differentiation of adult murine and human MABs have already been described in previous studies (Tonlorenzi *et al*, 2007; Diaz-Manera *et al*, 2010). Briefly, C57 nLacZ adult murine MABs, as well as human MABs, were plated on matrigel-coated dishes and at 80% of confluence cells were exposed to differentiation medium containing DMEM plus 2% horse serum and cultured for an additional 3–10 days before analysis.

Murine MABs were also incubated with BV11, non-related rat IgG, GGTI-298 or 007, and then used for the *in vitro* migration assays.

In experiments with living cells, blocking antibodies and the non-related IgG were used as purified immunoglobulins. Human cells had been obtained from muscle biopsies at Hospital San Raffaele, according to the protocol ‘Evaluation of regenerative properties of human mesoangioblasts’, submitted to the San Raffele Ethical Committee on January 08, 2007, approved on February 1 and authorized on February 20, 2007. All patients had signed informed consent.

### *In vivo* migration assay

In a first set of *in vivo* migration assays, 20 μl of 100 μM *Naja mossambica mossambica* cardiotoxin (Sigma-Aldrich) were injected directly into the gastrocnemius, tibialis anterior and quadriceps muscles of WT C57/BL6J, *PECAM-1*-null and *JAM-A*-null age-matched mice. Then, 24 h after cardiotoxin-induced damage, MABs were delivered via intra-arterial injections. In another set of *in vivo* experiments, *Sgca-*null and *Sgca*-null/scid/beige mice were injected into the tail vein with 3 mg/kg BV11 JAM-A neutralizing antibody, or non-related rat IgG (Del Maschio *et al*, 1999) 1 h prior to intra-arterial injections of MABs. In a separate experimental group, GGTI-298 (50 mg/kg resuspended in 1 ml vehicle per mouse) or vehicle (6% dimethyl sulfoxide, 0.6 mM dithiothreitol, normal saline solution) were given to *Sgca-*null mice via intra-peritoneal injections 1 h before intra-arterial MAB delivery. In the *Sgca-*null/ *JAM-A*-null mice, no treatments were performed before intra-arterial MAB injections. In all experiments, with the exception of functional experiments into *Sgca*-null/scid/beige (see Treadmill to exhaustion test), MABs have been injected into one femoral artery of each mouse, according to standard protocols (Tedesco *et al*, 2012). Mice were sacrificed 6 h after intra-arterial injections since previous studies established that within 6 h intra-arterially delivered MABs have completed transmigration from the vessel lumen into acutely or chronically damaged skeletal muscle (Galvez *et al*, 2006; Tedesco *et al*, 2012). In the case of *in vivo* engraftment experiments into *Sgca*-null/scid/beige mice, mice were sacrificed 3 weeks later. The hind limb muscles (gastrocnemius, tibialis and quadriceps) were collected and analysed.

### *In vitro* migration assay

*In vitro* migration assays were performed on murine ( *JAM-A*-WT, *JAM-A*-null) or human endothelial cells (HUVECs, with stable scrambled shRNA or *JAM-A* targeting shRNAs). Briefly, the cells were seeded on 8-μm transwell filters (Corning, NY, USA) that were previously coated with glutaraldehyde-crosslinked gelatin as follows: the culture supports were incubated for 1 h at RT with 0.5% (for murine endothelial cells) or 1.5% (for HUVECs) gelatin, followed by crosslinking with 2% glutaraldehyde solution (Sigma-Aldrich) for 15 min at RT. The glutaraldehyde was then replaced by 70% ethanol. After 1 h, 5 washes with sterile PBS were followed by an overnight incubation with PBS containing 2 mM glycine. Before cell seeding, the slides were washed 5 times with sterile PBS. Alternatively, μ-Dish^35 mm, high^ glass bottom (Ibidi) were coated with collagen gels (130 μl) containing Collagen R (Serva Electrophoresis GmbH, Heidelberg, Germany) and Collagen G that were prepared as previously described (Bauer *et al*, 2007). Endothelial cells were then grown as a monolayer to reach confluence in 72 h, in 5% CO_2_ at 37°C. When needed, the confluent cell monolayers were pre-incubated for 3 h with JAM-A neutralizing antibodies, or β1-integrin (10 μg/ml, (Noto *et al*, 1995)) or β3-integrin (3 μg/ml, (Ashkar *et al*, 2000)) or with the appropriate isotype-IgG as a control. Upon endothelium starvation, pre-treatments with GGTI-298, 007 or vehicle were performed for 30 min. Antibodies and chemical treatments were maintained during the assay. For extracellular matrix experiments, the confluent endothelial cell monolayers were extracted with 0.5% Triton X-100, followed by one rinse with 0.1 M NH_4_OH and three rinses with PBS (Giese *et al*, 1994) and the washed twice with DMEM containing 2% FBS.

MABs were labeled with 0.7 μM 6-carboxyfluorescin diacetate (6-CFDA) (Molecular Probes, Invitrogen, Eugene, OR, USA) for 20 min at 37°C. 3 × 10^4^ fluorescent murine or human MABs were added to the upper chamber and were left to migrate in 5% CO_2_ at 37°C for 6-8 h. After the scraping of the non-migrated cells on the upper face of the filters, the cells on the lower face were fixed with 4% paraformaldehyde for 10 min and washed in PBS. The migrated cells were counted under an inverted fluorescence microscope (AMG EVOS fl) using 10 × magnification. Five pictures of adjacent fields of the central zone of each filter were taken. The data were displayed as numbers of migrated cells per area.

### Adhesion assay

Confluent *JAM-A*-WT and *JAM-A*-null endothelial cells monolayers were grown in 96-well plate for 72 h. MABs were labeled with 0.7 μM 6-carboxyfluorescin diacetate (6-CFDA) (Molecular Probes, Invitrogen) for 20 min at 37°C before addition to endothelial cells monolayers (3 × 10^4^ cells/well). Co-cultures were left at 37°C for 30 min. Unbound MABs were removed and wells were washed with pre-warmed DMEM 0.1% BSA four times. Finally, adherent MABs were quantified using the fluorometer Wallac Victor3 1420 multilabel counter (Perkin Elmer, Whaltman, MA, USA). Wells without MABs were used to assay the background fluorescence. Wells with the starting amount of MABs were used to assay the total immunofluorescence. Each condition was done in triplicate.

### Production of lentiviruses and stable cell lines

shRNA against the human *JAM-A* gene and a non-targeting shRNA as a control were cloned into pLKO.1 puro-based lentiviral vectors and purchased from Sigma-Aldrich (St Louis, MO, USA). The *JAM-A* target sequences were listed in supplementary Table S1.

The cDNA coding for the wild-type mouse *PECAM-1* was kindly provided by Dr. Steven Albelda, Philadelphia, Pennsylvania. The mouse *PECAM-1* cDNA was amplified by PCR and subcloned into lentiviral expression vector pLVX-AcGFP-N1 (Clontech, Mountain View, CA, USA) using restrinction sites for SmaI and BstBI (Promega, Fitchburg, WI, USA). The primers used were listed in supplementary Table S1. The pLVX-Tdtomato-N1 (Clontech) plasmid was used to generate stable endothelial cells and MABs expressing the red fluorescent protein Tdtomato.

The lentiviral particles were produced using a four-plasmid transfection system as described previously (Taulli *et al*, 2005). Lentivirus-containing supernatants were collected 48 h and 72 h after transfection, passed through a 0.45 μM filter, and used in two consecutive cycles of infection of endothelial cells. About 48 h after the last infection, infected cells were selected with puromycin (3 μg/ml) for 72 h and maintained at 1.5 μg/ml puromycin.

### Immunoprecipitation and immunoblotting

For immunoprecipitation, confluent monolayers of endothelial cells were incubated with 80 g/ml dithiobis-(succinimidyl) propionate (Pierce, Rockford, IL, USA) for 30 min at 37°C. The cells were lysed in RIPA buffer (100 mM Tris–HCl, pH 7.4, 150 mM NaCl, 1% Triton, 1% deoxycholic acid, 0.1% SDS, 2 mM CaCl_2_, protease inhibitors [Roche] and phosphatase inhibitors [Sigma-Aldrich]) and JAM-A or EPAC-2 was immunoprecipitated overnight from 8 mg total extract, as described by Ebnet *et al* (2000). For total extracts preparation, the cells were harvested in Laemmli buffer (2.5% SDS, 20% glycerol, 0.125 M Tris-HCl, pH 6.8). Total extracts were incubated for 5 min at 100°C to allow protein denaturation, and then centrifuged for 5 min at 16 000 × *g* to discard cell debris. The supernatants were collected and the protein concentrations were determined using BC Protein Assay kits (Pierce), according to the manufacturer instructions.

Total extracts or the immunoprecipitated were loaded onto gels, separated by SDS-PAGE, transferred to a Protran Nitrocellulose Hybridization Transfer Membrane (0.2 μm pore size; Whatman). The membranes were probed with primary antibodies as indicated in the figures. Bound antibodies were visualized with the appropriate horseradish peroxidase-linked secondary antibody (1:2000; Cell Signaling Technology, Danvers, MA, USA) and specific binding was detected by the enhanced chemiluminescence (ECL) system (Amersham Biosciences, Uppsala, Sweden) using HyperfilmTM (Amersham Biosciences), or with SuperSignal West Femto Maximum Sensitivity Substrate kits (Thermo Pierce, Rockford, IL, USA) for chemiluminescence detection with a ChemiDoc XRS gel imaging system (Bio-Rad, Hercules, CA, USA). The molecular masses of proteins were estimated relative to the electrophoretic mobility of the cotransferred prestained protein marker, Broad Range (Cell Signaling Technology, Danvers, MA, USA). Densitometric analysis was performed using imagej Version 1.33 (National Institute of Health, Bethesda, MD, USA) or IMAGE LAB (Bio-Rad) software.

### Immunofluorescence microscopy

Immunofluorescence analyses were performed according to standard protocols (Lampugnani *et al*, 1997; Tedesco *et al*, 2012). Briefly, cells were cultured in 8-well μ-Slides (Ibidi, Martinsried, Germany). For MyHC staining, the cells were fixed with 4% paraformaldehyde for 10 min at 4°C. Once fixed, the cells were incubated for 30 min with 1% BSA, 0.2% Triton (TX-100) in PBS and for another 30 min with 10% donkey serum. For Sgca, β-gal and laminin staining of muscle tissue sections, the slices were not fixed but were directly blocked for 30 min with 1% BSA, 0.2% TX-100 in PBS, and for a further 30 min with 10% goat serum. For all of the other stainings, the cells were fixed and permeabilized in ice-cold methanol at −20°C, for 5 min. Fixed cells were incubated for 1 h in a blocking solution: for JAM-A and VE-cadherin staining, the blocking buffer was 2% BSA, 5% donkey serum and 0.05% Tween-20 in TBS. Cells and slices were then incubated overnight at 4°C or for 2 h at RT, with primary antibodies diluted in blocking buffer. The appropriate secondary antibodies (Alexa Fluor 488, 546, 594, 1:200; Invitrogen) were applied to the cells for 45 min at RT. Before mounting the cells, the cell nuclei were stained with 4′,6-diamidino-2-phenylindole (DAPI; 1:5000; Jackson ImmunoResearch, Westgrove, PA, USA) or Hoechst 33342 (1:1000; Fluka, Sigma-Aldrich), for 5 min at RT.

Samples were observed under a DMR fluorescence microscope (Leica, Solms, Germany) using 63 × or 100 × lenses, or a DMI 6000B fluorescence microscope (Leica) using 10 × or 20 × lenses. The images were captured using a charge-coupled camera, model 3, Hamamatsu or DFC 350FX, before processing through Adobe Photoshop and Adobe Illustrator. Only adjustments to brightness and contrast were used in the preparation of the figures. For comparison purposes, different sample images of the same antigen were acquired under constant acquisition settings.

For detection of apoptosis, Dead END™ FLUORIMETRIC TUNEL SYSTEM (Promega) has been used and immunofluorescence on sections has been performed following manufacturer's instructions.

Confocal microscopy was performed with a Leica TCS SP2 AOBS confocal microscope, equipped with yellow (Argon, 488 nm), red (561 nm solid state laser) and blue (633 nm HeNe laser) excitation laser lines. Image acquisition was performed using a 63 × /1.4 NA oil immersion objective (HCX PL APO 63 × Lbd Bl; Leica) and with spectral detection bands and scanning modalities optimized for the removal of channel cross-talk. Open-source software, IMAGEJ (Schneider *et al*, 2012), was used for the data analysis. Briefly, for the quantification of Fig 5D (right panel), we developed a macro for automated segmentation of the cells based on junctions definition by VE-cadherin. After background correction, the total fluorescence of JAM-A within each segmented area was measured. Finally, the mean fluorescences were calculated as the ratios of the total fluorescence signals to the number of pixels in the areas, expressed as arbitrary units.

### Live cell imaging and analysis

Time-lapse imaging using *JAM*-A-WT and *JAM*-A-null endothelial cells expressing Td-tomato and C57-GFP MABs on a Leica TCS SP5 confocal microscope equipped with a HCX Plan Apo 40× (1.25–0.75 NA) oil-immersion objective. A 488 argon laser, and a 561 diode laser have been used for the excitation of the GFP and Tdtomato respectively, setting the acquisition to sequential scanning to avoid cross-talk between the channels. The system is equipped with an incubator (OKOlab) to maintain 37°C in an atmosphere of 5% CO_2_. Stacks of images were taken every 7 min during a period up to 5 h, in optical planes separated by 1.5 μm along a *z-*axis range of 54 μm.

Time-lapse imaging using *JAM-A*-WT and *JAM-A*-null endothelial cells expressing PECAM-1-GFP was performed on a spinning disk confocal microscope (UltraVIEW VoX; PerkinElmer) equipped with a Plan Fluor 40× (1.30 NA) and Plan Apo VC 60x (1.40 NA) oil immersion objective (Nikon, Amsterdam, the Netherlands) and controlled by Volocity (Improvision, Perkin Elmer, MA, USA) software. The system was equipped with an environmental chamber (OKOlab, Naples, Italy) maintained at 37°C in an atmosphere of 5% CO_2_. For the endothelial dynamics (Fig 5B), stacks of images were taken every 12 min during a period up to 5 h, in optical planes (separated by 2 μm) along a *z* range of 12 μm. For the transmigration assay (Fig 5C), stacks of images were taken every 10 min during a period up to 5 h, in optical planes (separated by 2 μm) along a *z* range of 26 μm.

Movies were analysed using ImageJ by compiling images from the maximum projection of consecutive focal planes consistently within each experiment. Depth-coded stack were generated using the dedicated plug-in (Depth coded stack), which assigned a gradient of pseudo-colors ranging from red to blue, (see ‘Depth Index in Fig 5C), varying according to focal plane depth. 3-Dimentional reconstitutions of MABs through the endothelium were processed and edited using Volocity software (Perkin Elmer).

### Rap-1 activation assay and immunostaining

Activation of Rap-1 was measured *in vitro* on confluent murine endothelial cell monolayers. When needed, the cells were incubated with BV11 and the non-related rat IgG, or with TPA, 007 or GGTI-298, and then used for the assays. To detect *in vivo* Rap-1 activation, the hind limb muscles were collected and then processed by Tissue Lyser II (Qiagen, Hilden, Germany). The tissue lysates were cleared by centrifugation at 16 000 × *g* for 15 min and then used for the assays.

Rap-1 pull-down assays were performed using the GST-fused Rap-1-binding domain of Ral-GDS, which was obtained by transforming *Escherichia coli* strain BL21 with a pGEX-2T-Ral-GDS-RBD expression vector (Self *et al*, 2001). The fusion protein (GST-RalGDS-RBD) was affinity purified on glutathione-Sepharose 4B beads (GE Healthcare, Buckinghamshire, UK) using standard methods. The Rap-1 pull-down assays were performed as described previously (Franke *et al*, 1997). Rabbit polyclonal anti-Rap1 and anti-rabbit HRP-conjugated antibodies were used to reveal Rap-1-GTP and total Rap-1. Activated Rap-1 in ice-cold methanol fixed cells was detected by probing with the RBD from the Rap-1-GTP-interacting protein Ral-GDS-GST at 6 μg/ml, overnight at 4°C. A rabbit anti-GST antibody was used subsequently. Negative controls were performed as follows: by omitting the incubation with GST-RalGDS-RBD; by replacing GST-RalGDS-RBD with GST alone at the same dilution; by using the anti-GST antibody alone. To determine the amount of co-localization between Rap-1-GTP and VE-cadherin, we developed an in-house image-processing pipeline based on the MevisLAB platform (developed by MeVis Medical Solutions AG and Fraunhofer MEVIS, http://www.mevislab.de/). For each biological sample, three different images are input into the processing pipeline: an image depicting the localization of Rap-1-GTP (Rap-1-image), an imaging depicting the localization of VE-cadherin (VE-c image), and an image depicting the nucleus localization (DAPI-image). As a first step, the DAPI-image is thresholded (static threshold manually set to an intensity value of 30) and turned into a binary image, with nuclei set to 0 and the remaining pixels set to 1. Next, the VE-c image is de-noised by using an itk-based median filter of radius set to 1 pixel. The de-noised image is combined with the binary DAPI-image so that only the VE-c pixels not belonging to the nuclei are kept for further analysis. The Rap-1-image is processed in a similar way. The binary VE-c and Rap-1 images are finally used to assess co-localization. Only pixels set to 1 in both images are kept for further analysis, as these represent co-localization of VE-cadherin and Rap-1-GTP. The mean intensities of Rap-1-GTP co-localizing with VE-cadherin were finally calculated.

### Antibodies

All antibodies used are described in supplementary Table S2.

### Histology

Muscles were frozen in liquid nitrogen-cooled isopenthane and cryostat sections were used for histology. Tissue sections were stained with hematoxylin and eosin (Sigma-Aldrich) and/or X-Gal (Invitrogen) according to standard protocols (Tedesco *et al*, 2011).

### Intravenous injection of lysine-fixable cadaverine conjugated to Alexa Fluor-555

Cadaverine conjugated to Alexa Fluor-555 (3.125 mg/ml, in saline) was injected intravenously into the tail vein of adult (5 month old) mice (25 mg/kg). The circulation time was 2 h. For *in situ* detection of cadaverine, the anesthesized mice were perfused for 1-2 min with HBSS, followed by 5 min perfusion with 4% paraformaldehyde in PBS, pH 7.2. The organs were then removed and post-fixed in 4% paraformaldehyde at 4°C for 5-6 h. Images of dissected organs were captured using a stereomicroscope (SZX16, Olympus, Tokyo, Japan) equipped with a fluorescence long-pass filter for red fluorescent protein (excitation, 530–550 nm; emission, 575 nm). Image acquisition was performed using a 1 × objective with total magnification of 0.35 ×, supported by a RGB camera (Nikon Digital Sight DS-5Mc, Amsterdam, the Netherlands). The IMAGEJ open source software was used for data analysis. The mean fluorescences were calculated as the ratios of the total fluorescence signals to the number of pixels in the areas, expressed as arbitrary units.

**The paper explained**

**Problem**

Cell therapy is a promising route for the treatment of muscular dystrophies, severe and fatal genetic diseases of the skeletal muscle for which no cure is currently available. Injection of blood-vessel-associated stem cells, known as mesoangioblasts (MABs), has improved the regeneration of skeletal muscle and its function in different animal models of muscular dystrophies. Furthermore, donor human leukocyte antigen (HLA)-matched MABs are currently being transplanted into Duchenne Muscular Dystrophy patients in phase I/II clinical trial.

One potential limitation for cell therapy is the difficulty of ensuring that a sufficient number of reconstituting cells reach the damaged areas. Therefore, strategies to increase cell engraftment will be crucial to improve the successful outcome of such cell therapies. Although some efforts have been made towards the definition of the requirements for efficient engraftment of MABs, a deliberate protocol for the clinical application of this strategy is still missing.

**Results**

We identified the endothelial junctional protein JAM-A as a key regulator of MAB engraftment in regenerating skeletal muscle. We show that endothelial specific *JAM-A* gene inactivation or a JAM-A blocking antibody markedly enhance MAB engraftment *in vitro* and *in vivo* in mouse models. In searching for the mechanism of action, we observed that in absence of JAM-A, MABs of both murine and human origin can migrate more efficiently through endothelial cell monolayers. Furthermore, in the absence of JAM-A, the activation and junctional localization of the small GTPase Rap-1 is prevented due to the down-regulation of the cAMP-responsive Rap-1 guanine nucleotide exchange factors EPAC-1 and EPAC-2. This, in turn, inhibits junction tightening and allows MAB extravasation. Notably, pharmacological inhibition of Rap-1 significantly increased MAB engraftment *in vitro* and *in vivo*.

**Impact**

The identification of specific cues that steer stem cells from their niche and increase the efficiency of their engraftment to diseased tissues might help to identify tools to improve stem cell therapies. The data highlight the fact that targeting the endothelial cell-to-cell junctions may be a reasonable therapeutic strategy to enhance the engraftment of muscle cell progenitors (MABs) to dystrophic muscles. Furthermore, we identified Rap-1 chemical inhibitor as potential agent to optimize cell therapy protocols for muscular dystrophies.

These findings reveal an important role of the endothelium in the regulation of stem cell engraftment and will provide novel types of therapeutical intervention in the treatment of dystrophic patients.

### Quantitative real-time PCR analysis

RNA was isolated from muscles using TRIZOL (Invitrogen) and converted into double-stranded cDNA with the cDNA synthesis kit ImProm™-II Reverse Trascription System (Promega) according to the manufacturer instructions. qPCRs were performed with a M×3000P Stratagene thermocycler. For quantitative real time PCR analysis the primers used were listed in supplementary Table S1.

### Treadmill to exhaustion test

Exercise tolerance was tested on a treadmill (Columbus Instruments, Columbus, OH, USA) as previously reported (Tedesco *et al*, 2011, 2012). Untransplanted *Sgca*-null/scid/beige mice ( *n* = 3), *Sgca*-null/scid/beige mice transplanted with adult MABs ( *n* = 3) and *Sgca*-null/scid/beige mice transplanted with adult MABs and treated with GGTI-298 ( *n* = 3) were used for these studies. Briefly, mice were trained three times a week to the treadmill (10 min; speed of 6 m/min). Upon completion of the training protocol, mice have been tested for their motor capacity for 2 weeks, to have baseline measurements for each mouse. Transplantations were done bilaterally with 5 × 10^5^ adult MABs per femoral artery 24 h after the last exercise (baseline measurements). Three weeks after the transplantation mouse motor capacity was tested again on the treadmill. During the test mice were kept into a 10° inclined treadmill at speed of 6 meters/min and the speed was then augmented of 2 m/min every 2 min until exhaustion (when mice stop for more than 5 s without reengaging the treadmill). Data are shown as fold increase of motor capacity measured at different time points (21, 28 and 35 days post transplantation) and were normalized with the baseline performance (pre-transplant) of each mouse.

### Statistical analysis

Data were analyzed using GraphPad Prism 5 (La Jolla, CA, USA) or Excel, and the data are expressed as means ± standard error of the means (s.e.m.) or ± standard deviation (s.d.). Statistical significance was tested using student's two-tailed unpaired *t*-tests or one-way ANOVA. A probability of < 5% ( *P *< **0.05) was considered to be statistically significant.
