# Supplementary material for: Targeting endothelial junctional adhesion molecule-A/ EPAC/ Rap-1 axis as a novel strategy to increase stem cell engraftment in dystrophic muscles
Source: EMBO Mol Med. 2013 Dec 30;6(2):239–58. doi: 10.1002/emmm.201302520 (PMC3927958; doi:10.1002/emmm.201302520)
Supplement: Supplementary file 1 [file emmm0006-0239-sd1.pdf]

EMBO Molecular Medicine

Targeting Endothelial Junctional Adhesion Molecule-A/ EPAC/ Rap-1 axis as a Novel Strategy to Increase Stem-Cell Engraftment in Dystrophic Muscles

Monica Giannotta, Sara Benedetti, Francesco Saverio Tedesco, Monica Corada, Marianna Trani, Rocco D'Antuono, Queensta Millet, Fabrizio Orsenigo, Beatriz Gonzalez Galvez, Giulio Cossu, and Elisabetta Dejana

Corresponding author: Elisabetta Dejana, IFOM-FIRC Institute of Molecular Oncology

---

Review timeline:

Submission date:	17 January 2013
Editorial Decision:	07 February 2013
Revision received:	27 September 2013
Editorial Decision:	21 October 2013
Revision received:	05 November 2013
Accepted:	05 November 2013

Transaction Report:

(Note: With the exception of the correction of typographical or spelling errors that could be a source of ambiguity, letters and reports are not edited. The original formatting of letters and referee reports may not be reflected in this compilation.)

Editor: Céline Carret

1st Editorial Decision 07 February 2013

Thank you for the submission of your manuscript to EMBO Molecular Medicine. We have now heard back from the three referees whom we asked to evaluate your manuscript.

As you will see from the enclosed referees' comments, they all find the study to be relevant and of interest. However, they also raise a number of concerns that I would like you to address in a revised version of your manuscript.

All three referees are concerned with the lack of in vivo data: referees #1 and #2 insist upon showing improved muscle function following treatment and referee #3 would like to see data regarding JAM-A inhibition *in vivo*. We agree that these experiments would greatly improve the clinical significance of your data.

In addition, referee #1 and to a lesser extend referee #2 mention that the underlying mechanism should be elucidated. We strongly encourage developing the study as far as realistically possible in these direction to strengthen your findings.

While publication of the paper cannot be considered at this stage, I invite you to submit a revised manuscript addressing the Reviewers' concerns with additional experimental data where appropriate.

Please note that it is EMBO Molecular Medicine policy to allow a single round of revision only and

that, therefore, acceptance or rejection of the manuscript will depend on the completeness of your responses included in the next, final version of the manuscript.

As you know, EMBO Molecular Medicine has a "scooping protection" policy, whereby similar findings that are published by others during review or revision are not a criterion for rejection. However, I do ask you to get in touch with us after three months if you have not completed your revision, to update us on the status. Please also contact us as soon as possible if similar work is published elsewhere.

I look forward to seeing a revised form of your manuscript as soon as possible.

***** Reviewer's comments *****

Referee #1 (Comments on Novelty/Model System):

In the present well written and structured manuscript the authors provide new data regarding the role of the endothelial tight junction molecule JAM-A and JAM-A-induced intracellular signaling for the mesoangioblast homing to damaged muscles and their therapeutic effects. Indeed, the authors found that deficiency or inhibition of JAM-A in mice leads to an increased homing of mesoangioblasts to damaged muscles thereby supporting the differentiation of mesoangioblasts to muscle tissue. Additionally, the authors provide data that this is happening by an already known mechanism involving the JAM-A-mediated regulation of endothelial Rap1 activity. The data are interesting and novel and have potential translational value not only for skeletal muscle diseases but also may be relevant for the treatment of heart diseases. However, the data are incomplete in the present form. Hard data (with potential clinical relevance) regarding the effects of the treatments on the muscle functionality are missing. Moreover, the underlying mechanisms of action of the inhibition of endothelial JAM-A and Rap1 in the transmigration process of mesoangioblasts are also missing. Therefore, additional experiments are necessary to address these issues and to increase the potential medical impact of this manuscript.

Referee #1 (Remarks):

In the present well written and structured manuscript the authors provide new data regarding the role of the endothelial tight junction molecule JAM-A and JAM-A-induced intracellular signaling for the mesoangioblast homing to damaged muscles and their therapeutic effects. Indeed, the authors found that deficiency or inhibition of JAM-A in mice leads to an increased homing of mesoangioblasts in damaged muscles thereby supporting the differentiation of mesoangioblasts to muscles. Additionally, the authors provide data that this is happening by an already known mechanism involving the JAM-A-mediated regulation of endothelial Rap1 activity. The data are interesting and novel and have potential translational value not only for skeletal muscle diseases but also may be relevant for the treatment of heart diseases. However, the data are incomplete in the present form. Hard data (with potential clinical relevance) regarding the effects of the treatments on the muscle functionality are missing. Moreover, the underlying mechanisms of action of inhibition of endothelial JAM-A and Rap1 in the transmigration process of mesoangioblasts are not clear. Therefore, additional experiments are necessary to address these issues.

Major comments:
#1
The authors provide evidence that inhibition of endothelial JAM-A or Rap1 increases recruitment of mesoangioblasts to acutely and chronically damaged muscles thereby promoting their differentiation to muscle tissue as shown by immunofluorescence and histochemistry stainings. Even if these data suggest that inhibition of endothelial JAM-A or Rap1 might support muscle regeneration by mesoangioblasts, do not prove it, since data regarding the functionality of the muscles (which is clinically important) are missing. Specifically, in order to increase the evidence for the regeneration mediated by mesoangioblasts, the authors should study the effects of inhibition/deficiency of JAM-A and inhibition of Rap1 on the mesoangioblast-induced functional recovery of the muscles in vivo. These data will support the evidence for the translational potential of this approach.

#2
The authors provide data that mesoangioblasts transmigrate through endothelial cell monolayers and that endothelial JAM-A and Rap1 control/limit this process. However, the nature of the adhesive trans-interactions (between mesoangioblasts and endothelial cells) and the steps of this transmigration process are not known. Moreover, data regarding the mechanism by which JAM-A and Rap1 limit the transmigration process of mesoangioblasts are missing and it is unclear which steps of the transmigration process are affected by JAM-A deficiency and by inhibition of endothelial Rap1. Therefore, I suggest to the authors to study by employing real time microscopy:
a) the steps of the mesoangioblast transmigration process in relation to the JAM-A localization in endothelial cells by using fluorescent-labeled JAM-A-constructs in endothelial cells
b) to identify which steps of the transmigration process of the mesoangioblasts are affected by endothelial JAM-A deficiency and Rap1 inhibition

#3
Deficiency of JAM-A or inhibition of endothelial Rap1 does not only affect tight junctions but also, as demonstrated by other groups:
a) reduces the integrin-mediated adhesivity to extracellular cell matrix proteins. How can the authors exclude that inhibition of JAM-A or endothelial Rap1 does not affect transendothelial migration of MAB by an effect on endothelial integrins ?
b) induces tissue edema and reduces leukocyte infiltration (inflammation). Therefore, I suggest to the authors to study, whether these mechanisms could be involved in the therapeutic effects of inhibition of endothelial JAM-A and Rap1.

#4
Does an in vivo treatment with 007 reduce the homing and the regenerative capacity of MAB ?

Minor comments
#1
The effects on the subcellular localization of active RAP1 in the figure 5E are not obvious. Therefore, I suggest to the authors to provide better images assessing the localization of RAP1.

Referee #2 (Comments on Novelty/Model System):

This is an elegant study that addresses an important medical problem in dystrophy.

Referee #2 (Remarks):

Summary
The authors investigate the impact of JAM-A downregulation and JAM-A inhibitory antibodies on mesoangioblast engraftment in dystrophic muscle.

Concerns
1) The authors elegantly demonstrate that MABs reach the muscle tissue more efficiently in conditions of JAM-A deficiency or Rap1 inhibition. To demonstrate the functional relevance of these findings, it would be useful to complement the studies by demonstrating improved muscle function after manipulation of JAM-A or Rap1.
2) Please elaborate on the analysis in figure 5B. It is unclear why EPAC levels return to baseline at 15 hour after BV11 treatment. This is important as stable down-regulation of JAMA-A results in less EPAC. How many times were the experiments in figure 5B performed?
3) The use of a novel Rap1 inhibitor is an exciting approach with therapeutic potential. Can the authors please provide additional data to demonstrate the specificity of the inhibitor for Rap1.
4) The immunofluorescence images in Figure 5E are difficult to interpret. Please provide images showing more cells with high magnification inset. For quantifying active junctional Rap1 it would be useful if the fluorescence intensity is normalized to a protein signal that does not change e.g. total Rap1. Additionally, a merged image showing active Rap 1 and VE-cadherin could help in

highlighting loss of junctional Rap1.
5) Please revise or remove the co-immunoprecipitation of JAM-A and EPAC-2 since the current image is not convincing.
6) Please provide additional details related to the methods used for assessing MAB migration, such as the number of MABs used. Why was the 6 hour time point selected for the engraftment studies?
7) Please provide images showing histology of the adductor muscle in Figure 1B. Why is the muscle fiber size and spacing in the gastrocnemius of wild type and JAM-null mice different?
8) The authors inappropriately use a T-Test for data that has been normalized to a control (Figs 1A, 2A, 2B, 6F). Please use another statistical test or remove statements of significance.
9) Please comment on the mechanisms of MAB migration.

Referee #3 (Comments on Novelty/Model System):

As I explained in my review, I feel that additional experiments will be necessary to prove the overall message of this manuscript. Particularly, it is felt that data demonstrating the inhibition of the JAM-A axis in vivo following delivery of the inhibitor should be added.

Referee #3 (Remarks):

The manuscript from Giannotta et al. reports an increase in stem cell engraftment in dystrophic mice following blockade of JAM-A. The authors present a series of experiments in isolated cells as well as live mice indicating that mesoangioblasts from embryonic or adult origins are able to cross the endothelial barrier more efficiently when JAM-A and its downstream signals are inhibited in endothelial cells. While the findings in these studies are potentially promising and novel, it is felt that additional experiments will strengthen the conclusion that JAM-A blockade facilitates the migration of injected cells to the muscle tissue.

Major concerns:

In Figure 1, the authors do not show what the baseline engraftment of adult MAB is in WT mice, which serves as baseline control. It seems that adult cells engraft more efficiently than embryonic cells overall, so the appropriate reference control should be shown.

While genetic ablation of JAM-A in recipient mice indicates that engraftment of transplanted cells in the muscle tissue is increased, the data using BV11 inhibition in recipient mice prior to cell injection does not demonstrate that JAM-A and its downstream effectors are indeed inhibited, as this was not tested. The authors should prove that the inhibitor BV11 in vivo indeed blocks the JAM-A axis in recipient sarcoglycan null/beige mice.

The in vitro studies using downregulated JAM-A in HUVEC cells indicates that human cells migrate more easily when JAM-A expression is decreased. While these studies are supportive of the overall concept that cell migration might be increased when JAM-A is inhibited, they are not strictly supportive of the model proposed here. The authors inject human cells into mice, while the in vitro migration experiments are tested using human cells only. It is felt that a situation resembling more closely the in vivo model should be tested, as this would be more supportive.

The studies using GGTI-298 inhibitor in vivo suffer form the same limitations listed for the BV11 inhibitor. Indeed, effective in vivo blockade of JAM-A and downstream effectors should be supported by additional experiments, particularly given that this drug is delivered IP only 1 hr prior to MAB injection.

The immunoprecipitation of EPAC-2 and EPAC-1 using the JAM-A antibody seems very weak (particularly EPAC-2), given the amount of input material. Given that direct binding of these two proteins might be controversial, did the authors attempt a reverse pull-down?

1st Revision - authors' response 27 September 2013

Referee #1 (Comments on Novelty/Model System):

In the present well written and structured manuscript the authors provide new data regarding the role of the endothelial tight junction molecule JAM-A and JAM-A-induced intracellular signalling for the mesoangioblast homing to damaged muscles and their therapeutic effects. Indeed, the authors found that deficiency or inhibition of JAM-A in mice leads to an increased homing of mesoangioblasts to damaged muscles thereby supporting the differentiation of mesoangioblasts to muscle tissue. Additionally, the authors provide data that this is happening by an already known mechanism involving the JAM-A-mediated regulation of endothelial Rap1 activity. The data are interesting and novel and have potential translational value not only for skeletal muscle diseases but also may be relevant for the treatment of heart diseases. However, the data are incomplete in the present form. Hard data (with potential clinical relevance) regarding the effects of the treatments on the muscle functionality are missing. Moreover, the underlying mechanisms of action of the inhibition of endothelial JAM-A and Rap1 in the transmigration process of mesoangioblasts are also missing. Therefore, additional experiments are necessary to address these issues and to increase the potential medical impact of this manuscript.

We would like to thank Referee #1 for his/her favourable comments on our paper and for the recommendations, which helped us to complete our observations.

Referee #1 (Remarks):

Major comments:

#1

The authors provide evidence that inhibition of endothelial JAM-A or Rap1 increases recruitment of mesoangioblasts to acutely and chronically damaged muscles thereby promoting their differentiation to muscle tissue as shown by immunofluorescence and histochemistry stainings. Even if these data suggest that inhibition of endothelial JAM-A or Rap1 might support muscle regeneration by mesoangioblasts, do not prove it, since data regarding the functionality of the muscles (which is clinically important) are missing.

Following this Referee suggestion, we performed functional tests on dystrophic immunodeficient *Sgca*-null/scid/beige mice. In particular we assessed their relative motor capacity by means of treadmill to exhaustion test and the data are now shown in Fig 7G and discussed in the main text on page 16.

In details, we have performed functional measurements on three experimental groups:

- untransplanted *Sgca*-null/scid/beige mice
- *Sgca*-null/scid/beige mice injected bilaterally with $5x10^5$ cells intra-artery
- *Sgca*-null/scid/beige mice treated with GGTI-298 and injected bilaterally with $5x10^5$ cells intra-artery

The data show that treatment of *Sgca*-null/scid/beige mice with GGTI-298 prior mesoangioblasts intra-arterial injection improves their motor capacity (Fig 7G). Importantly, in this set of experiments we used a sub-optimal amount of cells ($5x10^5$ cells/IA) compared to what published before for functional tests (10^6 cells/IA; (Tedesco et al, 2012; Tedesco et al, 2011) in order to highlight the potential effect of GGTI-298.

We also added in Supporting Information Fig S6 data showing the trend of each age-matched group (2 months old; 4 months old; 7 months old). Values are plotted as means of motor capacity

(running time) obtained for each mouse at any time points (21 days, 28 days, 25 days post transplantation) and have been normalized on its own baseline (time to exhaustion before transplantation).

#2

The authors provide data that mesoangioblasts transmigrate through endothelial cell monolayers and that endothelial JAM-A and Rap1 control/limit this process. However, the nature of the adhesive trans-interactions (between mesoangioblasts and endothelial cells) and the steps of this transmigration process are not known. Moreover, data regarding the mechanism by which JAM-A and Rap1 limit the transmigration process of mesoangioblasts are missing and it is unclear which steps of the transmigration process are affected by JAM-A deficiency and by inhibition of endothelial Rap1. Therefore, I suggest to the authors to study by employing real time microscopy:

a) the steps of the mesoangioblast transmigration process in relation to the JAM-A localization in endothelial cells by using fluorescent-labelled JAM-A-constructs in endothelial cells

b) to identify which steps of the transmigration process of the mesoangioblasts are affected by endothelial JAM-A deficiency and Rap1 inhibition

As requested by this Referee, we investigated whether JAM-A acts on adhesion or transmigration of mesoangioblasts through endothelial cell monolayers. As now reported in the Supporting Information Fig S5A embryonic mesoangioblasts adhered in a similar manner to the endothelium regardless of the presence or the absence of JAM-A. Moreover, adult mesoangioblasts adhere even less to *JAM-A* null endothelial cells (Supporting Information Fig S5A). Thus, increased adhesion does not explain the different capacity of mesoangioblast to infiltrate through endothelial cell monolayers.

To further investigate the adhesion and transmigration process we performed live-cell imaging of adult mesoangioblasts (C57-GFP) migrating through *JAM-A*-WT and *JAM-A*-null endothelial cells. Both *JAM-A*-WT and *JAM-A*-null endothelial cells were stably infected to express the fluorescent protein Td-Tomato and the cells were seeded on collagen gels. This procedure facilitates the observation of mesoangioblasts crossing the endothelial monolayers and invading the matrix *in vitro*. We found that MABs make contact with the *JAM-A*-WT endothelial but they are unable cross the monolayer and retain a spherical shape up to 290 min. In contrast, MABs seeded on JAM-A-null endothelium are able to elongate pseudopods through the endothelial monolayer, transmigrate and invade the collagen gel (Fig 5A, Supporting Information Fig S5G and Movie M1).

To further test the hypothesis that endothelial junctions are less tight in absence of JAM-A, we made efforts to generate fluorescent-labelled JAM-A-constructs. We used different lentiviral vectors to express JAM-A protein as C- and N- terminal fusions of the AcGFP or Td-Tomato proteins and we also tried to introduce a flexible linker between the JAM-A and the fluorescent protein. Unfortunately, none of the constructs localized at the endothelial junctions strongly enough to be suitable for live-cell imaging experiments.

Therefore, to meet the Referee request, we performed live-cell imaging of both *JAM-A*-WT and null endothelial cells stably infected to express the AcGFP-tagged PECAM-1, in order to specifically follow the behaviour of the endothelial junctions.

During 5 h observation, we found that junctions in *JAM-A*-null endothelial monolayers are continuously formed and disrupted (Fig 5B and Supporting Information Movie M2), showing a more dynamic behaviour compared to *JAM-A*-WT endothelium. These data support the idea that mesoangioblasts transmigration through *JAM-A*-null endothelial cells is facilitated by the dynamic remodelling of the junctions (Fig 5C and Supporting Information Movie M3).

Finally, we performed a time course transmigration assay of C57-GFP mesoangioblasts through *JAM-A*-null endothelial cells. The junctions were stained with an anti-PECAM-1 antibody (Fig 5D). The orthogonal view and the 3D reconstruction showed that C57-GFP mesoangioblasts transmigrate across the endothelium via the paracellular route (Fig 5D) likely mediated by the local dynamic dismantling of the junctions described above. These data have been now included in the main text in a separate paragraph ("Changes in junction organization drive MAB transmigration through *JAM-A*-null endothelial cells " at pages 11-13).

#3

Deficiency of JAM-A or inhibition of endothelial Rap1 does not only affect tight junctions but also, as demonstrated by other groups:

a) reduces the integrin-mediated adhesivity to extracellular cell matrix proteins. How can the authors exclude that inhibition of JAM-A or endothelial Rap1 does not affect transendothelial migration of MAB by an effect on endothelial integrins?

b) induces tissue edema and reduces leukocyte infiltration (inflammation).

Therefore, I suggest to the authors to study, whether these mechanisms could be involved in the therapeutic effects of inhibition of endothelial JAM-A and Rap1.

a) Endothelial cells express mostly b1 and b3-integrins and the adhesive properties of these integrins may be affected by JAM-A and Rap-1 deficiency, as pointed out by this Referee (McSherry et al, 2011; Naik & Naik, 2006; Peddibhotla et al, 2013; Severson et al, 2009). We therefore studied whether the expression and function of these integrins may be altered in *JAM-A*-null endothelial cell monolayers. Western blot analysis showed that the absence of JAM-A results in a slight decrease of b1-integrin, as previously reported (McSherry et al, 2011; Severson et al, 2008; Severson et al, 2009). In contrast, the expression level of b3-integrin is increased in *JAM-A*-null endothelial cells compared to WT (Supporting Information Fig S5B, C). This modulation of b1 and b3-integrin expression levels, although quite small, suggests that there may be a differential organization of extracellular matrix proteins in JAM-A-null and WT endothelial cells. To test this possibility, we compared the subendothelial organization of fibronectin, laminin and collagen IV in *JAM-A*-null and WT endothelial cells (as now reported in Supporting Information Fig S5D) but we have been unable to detect significant differences.

In addition, we tested the migration of mesoangioblasts through the isolated matrix of JAM-A-null or WT endothelial cells. We prepared endothelial extracellular matrices by extracting cell monolayers seeded on Transwell filters, as described in Materials and Methods (Giese et al, 1994). These coated-filters were then used for the transmigration assay. We found that mesoangioblasts migrated in a comparable way across the filters coated with the extracellular matrix of *JAM-A*-null and WT cells (Supporting Information Fig 5SE).

Finally, to further investigate a possible role of endothelial b1 and b3-integrins in mesoangioblast transmigration we used specific integrin blocking antibodies (Ashkar et al, 2000; Noto et al, 1995). We found that inhibition of β1 and β3-integrins slightly increase (by 30%) transmigration through JAM-A-WT endothelial cells compared to non-related IgG (see Supporting Information Fig S5F). However, this increase is far less than the one we obtained with JAM-A neutralizing antibody (>80%, see Fig 3C, D) and, most importantly, mesoangioblast diapedesis through *JAM-A*-null endothelial cells was not significantly affected by the presence of b1 and b3-integrin blocking antibodies, compared to non-related IgG (Supporting Information Fig S5F).

Overall we believe that these new findings rule out a major involvement of b1 and b3-integrins in the increased transmigration of mesoangioblasts through JAM-A null endothelial cells. All these novel data are now included in Fig S5 and in the main text in a separate paragraph ("Changes in junction organization drive MAB transmigration through *JAM-A*-null endothelial cells" on pages 11-13).

b) To test for the possible presence of tissue edema when JAM-A (with BV11) or Rap-1 activation (with GGTI-298) are impaired, we performed *in vivo* experiments by injecting cadaverine-Alexa Fluor-555 (25 mg/kg) in the tail vein of *Sgca*-null mice treated with BV11 or GGTI-298. After 2 h the mice were sacrificed and lungs, livers, whole brains and muscles were collected for the quantification of fluorescence. The data did not show any significant accumulation of the tracer in the organs and muscles of both BV11- and GGTI-treated mice when compared with untreated (IgG or vehicle, respectively) mice (Supporting Information Fig S4A).

As requested, we also performed $CD68^+$ (macrophages) staining on *Sgca*-null/*JAM-A*-null and *Sgca*-null mice (Supporting Information Fig S3C). We found that the number of $CD68^+$ macrophages in *Sgca*-null/*JAM-A*-null mice was decreased compared with *Sgca*-null mice (Supporting Information Fig S3C, D), which is in agreement with previous data (Corada et al, 2005). To test whether the reduced number of macrophages could improve the engraftment of mesoangioblasts indirectly by increasing cell survival and reduce muscle damage, we performed TUNEL staining. We found that

apoptosis was not decreased in *Sgca*-null/*JAM-A*-null mice as compared to *Sgca*-null mice (Supporting Information Fig S3E, F).

These results are consistent with the observation that in all types of *in vitro* experiments, mesoangioblast diapedesis through JAM-A null endothelium is significantly increased also in absence of inflammation or tissue damage (Fig 3, Fig 4A-C and Fig 7C-E). The new data have been now included in the main text at pages 7-8).

#4

Does an in vivo treatment with 007 reduce the homing and the regenerative capacity of MAB?

007 has been used *in vitro* only or *in vivo* via intra-renal injections (Stokman et al, 2011). We discussed the possibility of using 007 *in vivo* with Johannes Bos who originally characterized this compound. However, he told us that the pharmacodynamics of 007 is not suitable for *in vivo* experiments because intravenously injection of this drug results in a very rapid clearance and the amount of the compound taken up by cells and tissues is very low.

To overcome this issue we used a modified form of 007, so called 007-AM, that should have an enhanced *in vitro* biological activity and an increased cellular uptake (Vliem et al, 2008). We tried different doses of 007-AM (2-3 mg/kg intravenously), but we could not observe a significant increase in Rap-1 activation *in vivo*. Therefore, a more extensive study of the pharmacokinetics of this drug *in vivo*, including the evaluation of its uptake by different types of cells and tissues is needed before reaching a correct dose regimen. We believe, however, that this would be far beyond the scope of this paper.

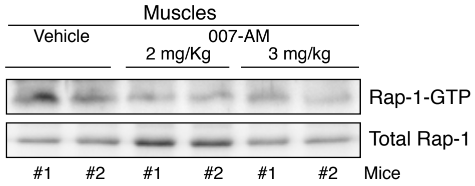

Sgca-null mice were treated with 007-AM (2-3 mg/Kg n = 2 for each dose) or with vehicle (n = 2) as control. After 2 h, the hind limb muscles (gastrocnemius, tibialis anterior and quadriceps) were collected and then processed by Tissue Lyser II. The protein extracts were incubated with the Ral-GDS-RBD probe. The active Rap-1 (Rap-1-GTP) and total Rap-1 were detected using an anti-Rap-1 antibody.

Minor comments

#1

The effects on the subcellular localization of active RAP1 in the figure 5E are not obvious. Therefore, I suggest to the authors to provide better images assessing the localization of RAP1.

To facilitate the interpretation of these data, we refined the panel E of the Fig 6 (previously named Fig 5E) including a new inset showing only the pixels deriving from active Rap-1 and VE-cadherin co-localization.

Referee #2 (Comments on Novelty/Model System):

This is an elegant study that addresses an important medical problem in dystrophy.

We warmly thank the Referee for his/her encouraging comments.

(Remarks):

Summary

The authors investigate the impact of JAM-A downregulation and JAM-A inhibitory antibodies on mesoangioblast engraftment in dystrophic muscle.

Concerns

The authors elegantly demonstrate that MABs reach the muscle tissue more efficiently in conditions of JAM-A deficiency or Rap1 inhibition. To demonstrate the functional relevance of these findings, it would be useful to complement the studies by demonstrating improved muscle function after manipulation of JAM-A or Rap1.

As also reported in the rebuttal to Referee #1, we performed functional tests by assessing mice motor capacity using a treadmill test to exhaustion and the data are now shown in Fig 7G and discussed in the main text on page 16.

In particular we tested three experimental groups:

- untreated *Sgca*-null/scid/beige mice intra-artery
- *Sgca*-null/scid/beige mice treated with $5x10^5$ cells intra-artery
- *Sgca*-null/scid/beige mice treated with $5x10^5$ cells and GGTI-298

The data show that treatment of *Sgca*-null/scid/beige mice with GGTI-298 prior mesoangioblast intra-arterial injection improves their motor capacity (Fig 7G). Importantly, in these set of experiments we used a sub-optimal amount of cells ($5x10^5$ cells) compared to what published before for functional tests (10^6 cells, Tedesco et al, 2012; Tedesco et al, 2011) in order to highlight the potential effect of GGTI-298. Values in Fig 7G, are plotted as means of motor capacity (running time) obtained for each mouse at any time points (21 days, 28 days, 25 days post transplantation) and have been normalized on its own baseline (time to exhaustion before transplantation).

We also added in Supporting Information Fig S6 data showing the trend of each age-matched group (2 months old; 4 months old; 7 months old).

Please elaborate on the analysis in figure 5B. It is unclear why EPAC levels return to baseline at 15 hour after BV11 treatment. This is important as stable down-regulation of JAM-A results in less EPAC. How many times were the experiments in figure 5B performed?

In the manuscript we reported that stable down-regulation of JAM-A results in less EPAC-1 and EPAC-2 (Fig 6A, previously named 5A). In the attempt to modulate EPAC-1 and EPAC-2 expression by acute inhibition of JAM-A, we performed a time course experiment in the presence of BV11 antibody. In Fig 6B we showed that EPAC-1 and EPAC-2 expression decreases but returns to baseline within 15 hours after BV11 treatment. A possible explanation is that BV11 loses its activity after 15 hours. To test this possibility, we followed JAM-A localization during the 15 hours duration of the experiment. Eight hours after BV11 treatment, JAM-A staining appeared more diffuse and less intense at the junctions while at 15 hours junctional staining of JAM-A was restored. These observations are consistent with restoration of EPAC-2 expression reported in the figure. We include these data for the Referee perusal.

The experiment reported in Fig 6B page 39 is representative of three independent experiments as it is now reported in the legend to the Figure.

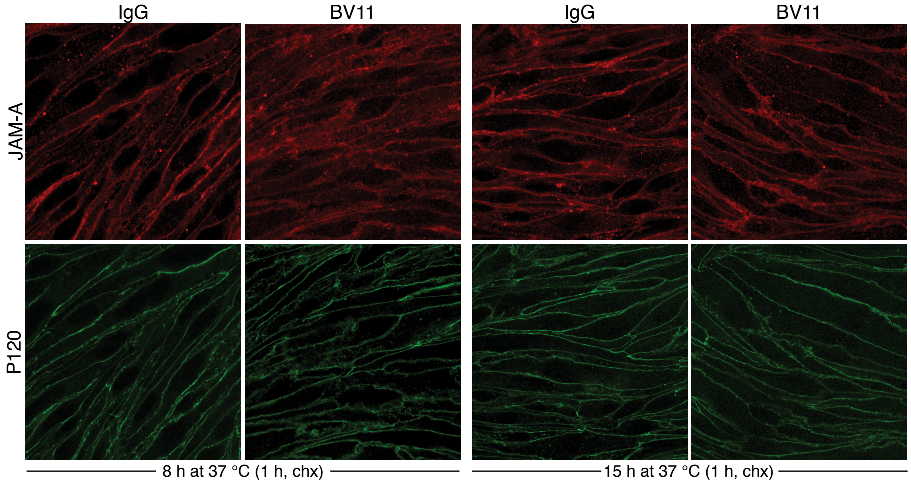

Confluent monolayers of endothelial cells were treated with non-related IgG, and BV11 (20 µg/ml) at 37 °C, for the indicated times. The cells were then incubated for the last 1 h with 100 µg/ml cycloheximide (chx) to inhibit protein synthesis, fixed and stained for JAM-A (red) and p120 (marker for junctions, green).

The use of a novel Rap1 inhibitor is an exciting approach with therapeutic potential. Can the authors please provide additional data to demonstrate the specificity of the inhibitor for Rap1.

We thank the Referee for this comment but GGTI-298 is not a novel Rap-1 inhibitor. It was used previously in a number published studies as a specific Rap-1 inhibitor (Chen & Dickman, 2005; De Grandis et al, 2013; Funaki et al, 2010; Hecquet et al, 2002; Kanda & Watanabe, 2007; Kogut et al, 2007; Lee et al, 2011; Li et al, 2010). The dose of GGTI-298 that we used in our experiments is consistent with the ones previously published to specifically inhibit Rap-1 (Kanda & Watanabe, 2007; Kim et al, 2010; Kogut et al, 2007).

The immunofluorescence images in Figure 5E are difficult to interpret. Please provide images showing more cells with high magnification inset. For quantifying active junctional Rap1 it would be useful if the fluorescence intensity is normalized to a protein signal that does not change e.g. total Rap1. Additionally, a merged image showing active Rap 1 and VE-cadherin could help in highlighting loss of junctional Rap1.

As suggested by the Referee, we have now included lower magnification images and a new inset showing the pixels deriving from active Rap-1 and VE-cadherin co-localization (Fig 6E, previously named Fig 5E). Unfortunately, the available antibodies directed to total Rap1 do not work well in immunofluorescence as far as our experience.

Please revise or remove the co-immunoprecipitation of JAM-A and EPAC-2 since the current image is not convincing.

We revised the co-immunoprecipitation of JAM-A. In Fig 6G, to avoid saturation of the input and to improve protein detection in IP we now show bands obtained using different exposure times. Furthermore, to strengthen this finding we performed a reverse co-immunoprecipitation of EPAC-2 and we confirmed that JAM-A forms a complex with EPAC-2 and EPAC-1. These data are now included in Fig 6, panel H.

Please provide additional details related to the methods used for assessing MAB migration, such as the number of MABs used. Why was the 6 hour time point selected for the engraftment studies?

We thank the Referee for giving us the opportunity to clarify to the readers some important technical details. For *in vivo* transmigration assays we injected cells into the femoral artery following a procedure described by (Tedesco et al, 2011).

In all sets of *in vivo* experiments, we used murine mesoangioblasts previously transduced with GFP- or nLacZ-lentiviral vectors (Supporting Information Fig S2) in order to be able to trace them once infiltrating in the murine muscles.

As specified in Materials and Methods section on page 22, 6 hours after intra-arterial injection, mice were sacrificed and hind limb muscles (tibialis anterior, gastrocnemius and quadriceps) were collected.

We used a 6-hour time point for transmigration analysis since we know from previous work (Galvez et al, 2006; Tedesco et al, 2012) that intra-arterially delivered mesoangioblasts have completed transmigration from the vessel lumen into acutely or chronically damaged skeletal muscle.

In vivo transmigration was evaluated by real time PCR of nLacZ (Fig 1A, 2A, 2B and 7F) or GFP (Fig 1A).

In the case of nLacZ-injected mesoangioblasts, engraftment was also evaluated by immunofluorescence (using an anti-b-gal antibody) or X-gal staining and by counting the positive cells on transversal cryosections (Fig 1C, 1D, 2C and 2D).

We added these details where appropriate i.e. figure legends and/or Materials and Methods section on page 22.

Please provide images showing histology of the adductor muscle in Figure 1B. Why is the muscle fibre size and spacing in the gastrocnemius of wild type and JAM-null mice different?

Regarding experiment of Fig 1, cardiotoxin was not directly injected in the adductor muscles but, as specified in Materials and Methods section (page 22), only in tibialis anterior, gastrocnemius and quadriceps (that in the manuscript we grouped together under the nomenclature “hind limb muscles”). Taking into account the Referee comment and after careful consideration, we decided that images of the adductor muscle, shown in Fig 1B of the original version of the manuscript might be misleading and we decided to remove them. We now show engraftment only in hind limb cardiotoxin-treated muscles (tibialis anterior, gastrocnemius and quadriceps).

We agree that, in the original figure, fibre size and spacing appear different in *JAM-A*-null and WT mice. This is likely due to the fact that cardiotoxin does not always induce a reproducible damage. In the present Fig 1C we changed Xgal+H&E images with others, which are more easily comparable and we added in Fig 1 B, H&E images from *JAM-A*-null and WT mice (Fig 1B) showing a cardiotoxin damage quite similar in both mouse strains.

Importantly, to avoid variability related to cardiotoxin damage, in the present work we also applied other approaches, i.e. treatment of dystrophic *Sgca*-null mice with anti-JAM-A neutralizing antibody or GGTI and mesoangioblast engraftment in *Sgca*-null/*JAM-A*-null mice (Fig 2).

The authors inappropriately use a T-Test for data that has been normalized to a control (Figs 1A, 2A, 2B, 6F). Please use another statistical test or remove statements of significance.

As recommended by this Referee we added an additional figure (in Supporting Information Fig S1) were we show all the raw data we obtained for all experiments in Fig 1A, 2A, 2B and 7F (previously named 6F). On these raw data we performed statistical analysis (T-test) and we show in the main figures only the relative fold increases (Fig 1A, Fig 2A, B and Fig 7F).

Please comment on the mechanisms of MAB migration.

We have now included additional data reported in the new Fig 5, panels A-D and described in the main text in the paragraph “Changes in junction organization drive MAB transmigration through *JAM-A*-null endothelial cells” on pages 11-13), which clarify several aspects of mesoangioblast transmigration through JAM-A-WT and *JAM-A*-null endothelial cells.

Referee #3 (Comments on Novelty/Model System):

As I explained in my review, I feel that additional experiments will be necessary to prove the overall message of this manuscript. Particularly, it is felt that data demonstrating the inhibition of the JAM-A axis in vivo following delivery of the inhibitor should be added.

Referee #3 (Remarks):

The manuscript from Giannotta et al. reports an increase in stem cell engraftment in dystrophic mice following blockade of JAM-A. The authors present a series of experiments in isolated cells as well as live mice indicating that mesoangioblasts from embryonic or adult origins are able to cross the endothelial barrier more efficiently when JAM-A and its downstream signals are inhibited in endothelial cells. While the findings in these studies are potentially promising and novel, it is felt that additional experiments will strengthen the conclusion that JAM-A blockade facilitates the migration of injected cells to the muscle tissue.

Major concerns:

In Figure 1, the authors do not show what the baseline engraftment of adult MAB is in WT mice, which serves as baseline control. It seems that adult cells engraft more efficiently than embryonic cells overall, so the appropriate reference control should be shown.

We previously showed the “Fold increase of MAB engraftment in hind limb muscles of null *vs* WT mice” (y axis label) and we used the X axis started from 1 as baseline, which is the value of engraftment we set for our WT mice in all the different and independent experiments. To simplify we now show columns relative to the baseline engraftment in the bar graph (Fig 1A).

Importantly, we added an additional figure (in Supporting Information Fig S1) were we show all the raw data we obtained for all experiments in Fig 1A, 2A, 2B and 7F (previously named 6F). On these raw data we performed statistical analysis (T-test) and we show in the main figures only their relative fold increases (Fig 1A, Fig 2A, B and Fig 7F).

While genetic ablation of JAM-A in recipient mice indicates that engraftment of transplanted cells in the muscle tissue is increased, the data using BV11 inhibition in recipient mice prior to cell injection does not demonstrate that JAM-A and its downstream effectors are indeed inhibited, as this was not tested. The authors should prove that the inhibitor BV11 in vivo indeed blocks the JAM-A axis in recipient sarcoglycan null/beige mice.

In previously published studies (Del Maschio et al, 1999; Martin-Padura et al, 1998) BV11 was found to selectively inhibit JAM-A *in vivo* and *in vitro*. To further prove that BV11 inhibits JAM-A activity *in vivo* also in our experimental settings, we evaluated the activity of its downstream effector Rap-1. To this aim, we performed a pull-down assays for active GTP-loaded Rap-1 in muscle extracts of mice treated with the antibody. We found that Rap-1 activation was decreased upon BV11 treatment in the hind limb muscles of sarcoglycan null/beige mice. These data are now included in Supporting Information Fig S4B.

The in vitro studies using down regulated JAM-A in HUVEC cells indicates that human cells migrate more easily when JAM-A expression is decreased. While these studies are supportive of the overall concept that cell migration might be increased when JAM-A is inhibited, they are not strictly supportive of the model proposed here. The authors inject human cells into mice, while the in vitro migration experiments are tested using human cells only. It is felt that a situation resembling more closely the in vivo model should be tested, as this would be more supportive.

In our experiments we always used an intra-specific setting, i.e. *in vitro* transmigration experiments have been performed using murine mesoangioblasts and murine endothelial cells coming from *JAM-A*-null or WT mice (Fig 3 and Fig 7 C, D). Therefore, *in vivo* experiments reproduce *in vitro* conditions i.e. murine mesoangioblasts injected into murine models of acute (Fig 1) or chronic muscle injury (Fig 2 and Fig 7F).

To test whether the same phenomenon occurred in human cells, we performed trans-migration experiments *in vitro* with human mesoangioblasts and human endothelium (Fig 4 and Fig 7E) and the results confirmed the data obtained in mice.

The studies using GGTI-298 in vivo suffer form the same limitations listed for the BV11 inhibitor. Indeed, effective in vivo blockade of JAM-A and downstream effectors should be supported by additional experiments, particularly given that this drug is delivered IP only 1 hr prior to MAB injection.

As suggested by the Referee, we tested the ability of GGTI-298 to inhibit Rap-1 in our experimental setting. We performed a pull-down assay for active GTP-loaded Rap-1. We found a reduction of Rap-1 activation in the hind limb muscles of sarcoglycan null/beige mice treated with GGTI-298 as compared to control mice.

These data are now included in Supporting Information Fig S4B

The immunoprecipitation of EPAC-2 and EPAC-1 using the JAM-A antibody seems very weak (particularly EPAC-2), given the amount of input material. Given that direct binding of these two proteins might be controversial, did the authors attempt a reverse pull-down

We revised the co-immunoprecipitation of JAM-A as suggested. Indeed, in Fig 6G, to avoid saturation of the input and to improve protein detection of immunoprecipitated proteins we showed bands obtained using different exposure times. Moreover, as suggested, to strengthen this finding we performed a reverse co-immunoprecipitation of EPAC-2 and we confirmed that JAM-A forms a complex with EPAC-2 and EPAC-1. These data have been now included in Fig 6, panel H.

REFERENCES

Ashkar S, Weber GF, Panoutsakopoulou V, Sanchirico ME, Jansson M, Zawaideh S, Rittling SR, Denhardt DT, Glimcher MJ, Cantor H (2000) Eta-1 (osteopontin): an early component of type-1 (cell-mediated) immunity. Science (New York, N.Y 287: 860-864

Chen C, Dickman MB (2005) cAMP blocks MAPK activation and sclerotial development via Rap-1 in a PKA-independent manner in Sclerotinia sclerotiorum. Mol Microbiol 55: 299-311

Corada M, Chimenti S, Cera MR, Vinci M, Salio M, Fiordaliso F, De Angelis N, Villa A, Bossi M, Staszewsky LI et al (2005) Junctional adhesion molecule-A-deficient polymorphonuclear cells show reduced diapedesis in peritonitis and heart ischemia-reperfusion injury. Proc Natl Acad Sci U S A 102: 10634-10639

De Grandis M, Cambot M, Wautier MP, Cassinat B, Chomienne C, Colin Y, Wautier JL, Le Van Kim C, El Nemer W (2013) JAK2V617F activates Lu/BCAM-mediated red cell adhesion in polycythemia vera through an EpoR-independent Rap1/Akt pathway. Blood 121: 658-665

Del Maschio A, De Luigi A, Martin-Padura I, Brockhaus M, Bartfai T, Fruscella P, Adorini L, Martino G, Furlan R, De Simoni MG et al (1999) Leukocyte recruitment in the cerebrospinal fluid of mice with experimental meningitis is inhibited by an antibody to junctional adhesion molecule (JAM). J Exp Med 190: 1351-1356

Funaki C, Hodges RR, Dartt DA (2010) Identification of the Raf-1 signaling pathway used by cAMP to inhibit p42/p44 MAPK in rat lacrimal gland acini: role in potentiation of protein secretion. Invest Ophthalmol Vis Sci 51: 6321-6328

Galvez BG, Sampaolesi M, Brunelli S, Covarello D, Gavina M, Rossi B, Constantin G, Torrente Y, Cossu G (2006) Complete repair of dystrophic skeletal muscle by mesoangioblasts with enhanced migration ability. J Cell Biol 174: 231-243

Giese A, Rief MD, Loo MA, Berens ME (1994) Determinants of human astrocytoma migration. Cancer Res 54: 3897-3904

Hecquet C, Lefevre G, Valtink M, Engelmann K, Mascarelli F (2002) cAMP inhibits the proliferation of retinal pigmented epithelial cells through the inhibition of ERK1/2 in a PKA-independent manner. Oncogene 21: 6101-6112

Kanda Y, Watanabe Y (2007) Adrenaline increases glucose transport via a Rap1-p38MAPK pathway in rat vascular smooth muscle cells. Br J Pharmacol 151: 476-482

Kim SP, Ha JM, Yun SJ, Kim EK, Chung SW, Hong KW, Kim CD, Bae SS (2010) Transcriptional activation of peroxisome proliferator-activated receptor-gamma requires activation of both protein kinase A and Akt during adipocyte differentiation. Biochem Biophys Res Commun 399: 55-59

Kogut MH, Genovese KJ, He H (2007) Flagellin and lipopolysaccharide stimulate the MEK-ERK signaling pathway in chicken heterophils through differential activation of the small GTPases, Ras and Rap1. Mol Immunol 44: 1729-1736

Lee DH, Lee HR, Shin HK, Park SY, Hong KW, Kim EK, Bae SS, Lee WS, Rhim BY, Kim CD (2011) Cilostazol enhances integrin-dependent homing of progenitor cells by activation of cAMP-dependent protein kinase in synergy with Epac1. J Neurosci Res 89: 650-660

Li L, Wang S, Jezierski A, Moalim-Nour L, Mohib K, Parks RJ, Retta SF, Wang L (2010) A unique interplay between Rap1 and E-cadherin in the endocytic pathway regulates self-renewal of human embryonic stem cells. Stem Cells 28: 247-257

Martin-Padura I, Lostaglio S, Schneemann M, Williams L, Romano M, Fruscella P, Panzeri C, Stoppacciaro A, Ruco L, Villa A et al (1998) Junctional adhesion molecule, a novel member of the immunoglobulin superfamily that distributes at intercellular junctions and modulates monocyte transmigration. J Cell Biol 142: 117-127

McSherry EA, Brennan K, Hudson L, Hill AD, Hopkins AM (2011) Breast cancer cell migration is regulated through junctional adhesion molecule-A-mediated activation of Rap1 GTPase. Breast Cancer Res 13: R31

Naik MU, Naik UP (2006) Junctional adhesion molecule-A-induced endothelial cell migration on vitronectin is integrin alpha v beta 3 specific. J Cell Sci 119: 490-499

Noto K, Kato K, Okumura K, Yagita H (1995) Identification and functional characterization of mouse CD29 with a mAb. Int Immunol 7: 835-842

Peddibhotla SS, Brinkmann BF, Kummer D, Tuncay H, Nakayama M, Adams RH, Gerke V, Ebnet K (2013) Tetraspanin CD9 links junctional adhesion molecule-A to alphavbeta3 integrin to mediate basic fibroblast growth factor-specific angiogenic signaling. Mol Biol Cell 24: 933-944

Severson EA, Jiang L, Ivanov AI, Mandell KJ, Nusrat A, Parkos CA (2008) Cis-dimerization mediates function of junctional adhesion molecule A. Mol Biol Cell 19: 1862-1872

Severson EA, Lee WY, Capaldo CT, Nusrat A, Parkos CA (2009) Junctional adhesion molecule A interacts with Afadin and PDZ-GEF2 to activate Rap1A, regulate beta1 integrin levels, and enhance cell migration. Mol Biol Cell 20: 1916-1925

© EMBO

Stokman G, Qin Y, Genieser HG, Schwede F, de Heer E, Bos JL, Bajema IM, van de Water B, Price LS (2011) Epac-Rap signaling reduces cellular stress and ischemia-induced kidney failure. J Am Soc Nephrol 22: 859-872

Tedesco FS, Gerli MF, Perani L, Benedetti S, Ungaro F, Cassano M, Antonini S, Tagliafico E, Artusi V, Longa E et al (2012) Transplantation of Genetically Corrected Human iPSC-Derived Progenitors in Mice with Limb-Girdle Muscular Dystrophy. Sci Transl Med 4: 140ra189

Tedesco FS, Hoshiya H, D'Antona G, Gerli MF, Messina G, Antonini S, Tonlorenzi R, Benedetti S, Berghella L, Torrente Y et al (2011) Stem cell-mediated transfer of a human artificial chromosome ameliorates muscular dystrophy. Sci Transl Med 3: 96ra78

Vliem MJ, Ponsioen B, Schwede F, Pannekoek WJ, Riedl J, Kooistra MR, Jalink K, Genieser HG, Bos JL, Rehmann H (2008) 8-pCPT-2'-O-Me-cAMP-AM: an improved Epac-selective cAMP analogue. Chembiochem 9: 2052-2054

2nd Editorial Decision 21 October 2013

Thank you for the submission of your revised manuscript to EMBO Molecular Medicine. We have now received the enclosed reports from the referees that were asked to re-assess it. As you will see the reviewers are now fully supportive and I am pleased to inform you that we will be able to accept your manuscript pending the following final amendments:

- We would like to encourage you to follow referee’s 2 advice and add the newly added important information in the abstract of the article as this will undoubtedly make it more attractive to the readers.

- We would also recommend to follow this referee's second point and provide better western blot images. In fact, as you certainly know, we now encourage the the publication of source data, particularly for electrophoretic gels and blots, with the aim of making primary data more accessible and transparent to the reader. Would you be willing to provide a PDF file per figure that contains the original, uncropped and unprocessed scans of all or key gels used in the figures? The PDF files should be labeled with the appropriate figure/panel number, and should have molecular weight markers; further annotation may be useful but is not essential. The PDF files will be published online with the article as supplementary "Source Data" files. If you have any questions regarding this just contact me.

Please submit your revised manuscript within two weeks. I look forward to seeing a revised form of your manuscript.

***** Reviewer's comments *****

Referee #1 (Remarks):

No further comments.

Referee #2 (Remarks):

The revised version of this manuscript is significantly improved. The additional data provides a much better understanding of the role of JAM-A in regulating trans-endothelial migration of mesanglioblasts, and show the significance of MAB engraftment in muscle function. Inhibition of endothelial JAM-A may indeed offer an improved therapy muscular dystrophies.

However, few minor details still remain to address.

1. The authors should amend the abstract for the study to reflect additional important findings that were added to the revised version. This should include the role for JAM in MAB migration and importantly, the improved muscle function following JAM-A inhibition.
2. The blots from IP experiments in Fig 6G and H are not of sufficient quality for publication. Can the authors improve the quality of these blots?

Referee #3 (Comments on Novelty/Model System):

The revised version of this paper is very much improved. The findings are innovative and the experiments support the conclusions provided by the authors.

Referee #3 (Remarks):

The authors have done an outstanding job in addressing all of my previous concerns. Overall, this work is highly innovative and of high impact in the field. I have no additional concerns.

2nd Revision - authors' response 05 November 2013

As you and Referee 2 suggest, we added in the Abstract two sentences to highlight the novel results now reported in our paper.

Panels 6 G and H report a representative image of the co-immunoprecipitation experiments that we repeated several times with consistent results. As requested we provide a better quality Western blot images adjusting brightness and contrast and we believe that this is the best result we can obtain. The bands corresponding to EPAC-1 and 2, although weak are detectable. Furthermore, as requested by the Referee 3, we detected the interaction between JAM-A and EPAC-1 and 2 immunoprecipitating with either JAM-A or EPAC-2 antibodies. Indeed, Referee 3, who previously raised the same problem, appears satisfied by the new panel H presented. It is conceivable that the complex of JAM-A with EPAC-1 and 2 engage only a relatively small number of molecules taking into account the multiple roles of both EPACs and JAM-A. We believe that these data constitute a nice addition to our story.

However, in case you still feel that these two panels cannot be published in the Journal, and taking into account that they are not indispensable for the overall message of the paper, we are open to drop these results following Referee 2 previous suggestion. Panel A of Figure 4 has been provided at better resolution for publication.

As requested, we provided a PDF file per figure that contains the original, uncropped and unprocessed scans of all gels used in the figures.
