# Supplementary figures and images for: Targeting endothelial junctional adhesion molecule-A/ EPAC/ Rap-1 axis as a novel strategy to increase stem cell engraftment in dystrophic muscles
Source: EMBO Mol Med. 2013 Dec 30;6(2):239–58. doi: 10.1002/emmm.201302520 (PMC3927958; doi:10.1002/emmm.201302520)

Giannotta et al., Figure 4

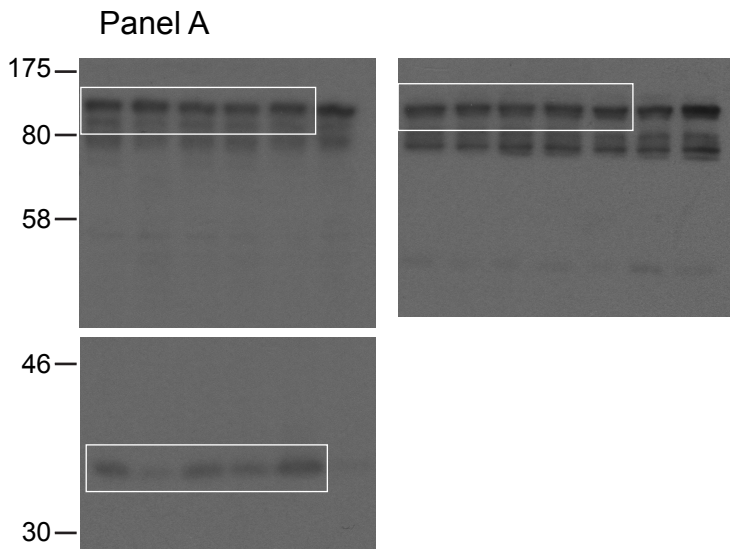

Supplement: Supplementary file 2 [file emmm0006-0239-sd2.pdf]

Panel A

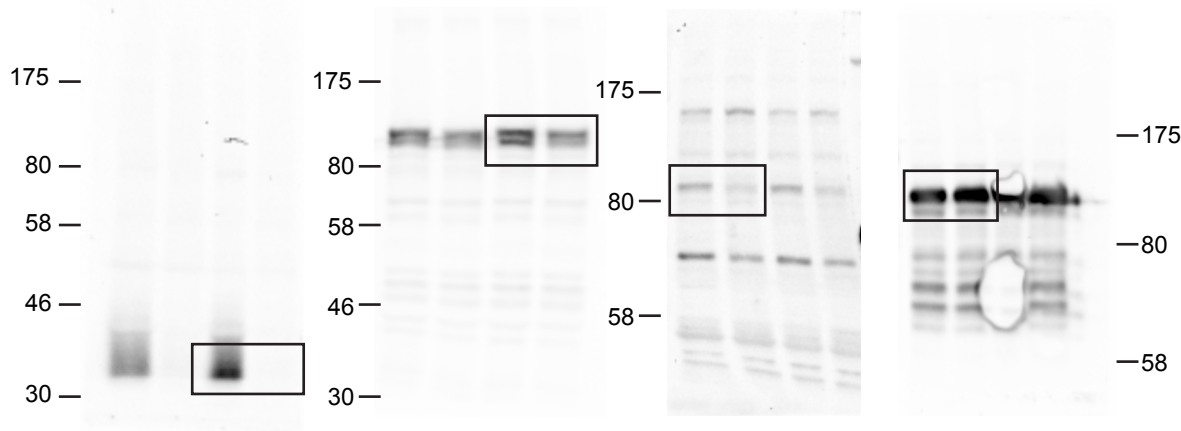

Panel B

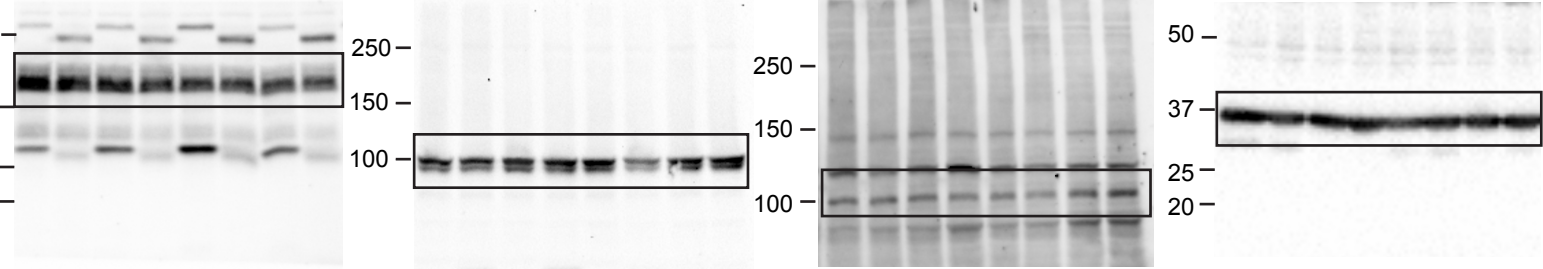

Panel C

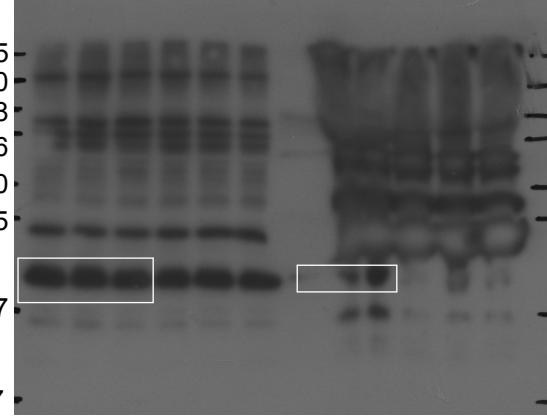

Panel D

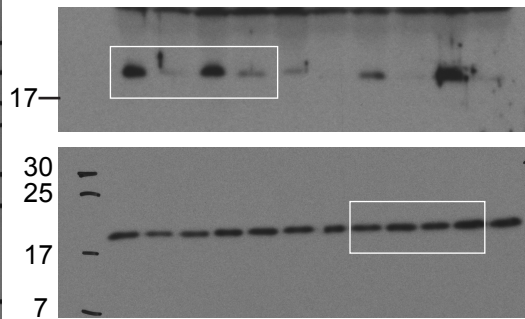

Panel G

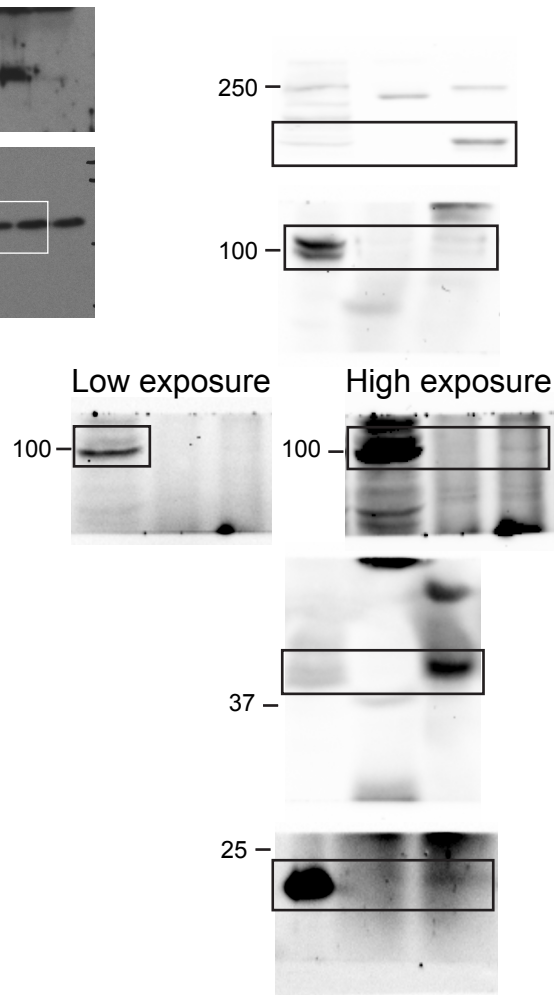

Panel H

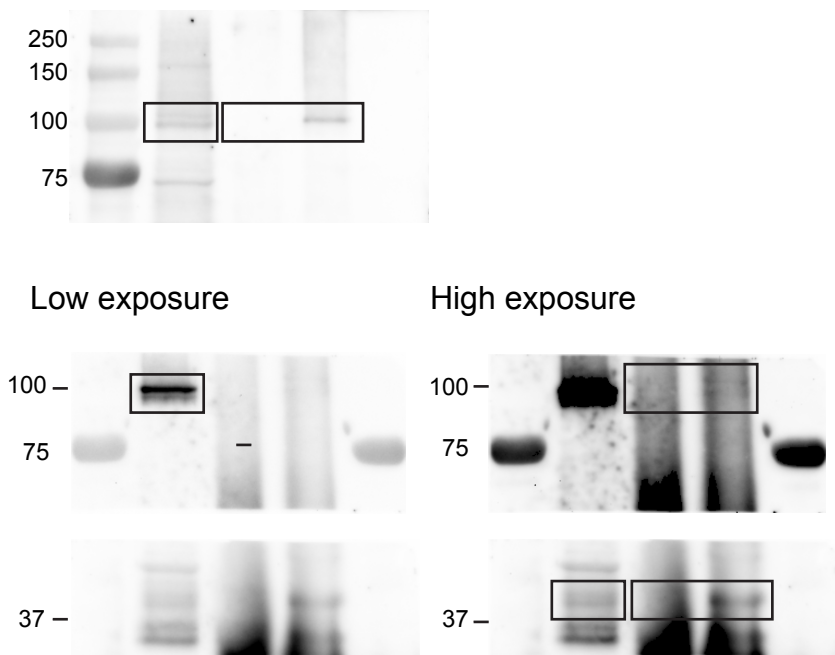

Supplement: Supplementary file 3 [file emmm0006-0239-sd3.pdf]

Giannotta et al., Figure 7

Panel A

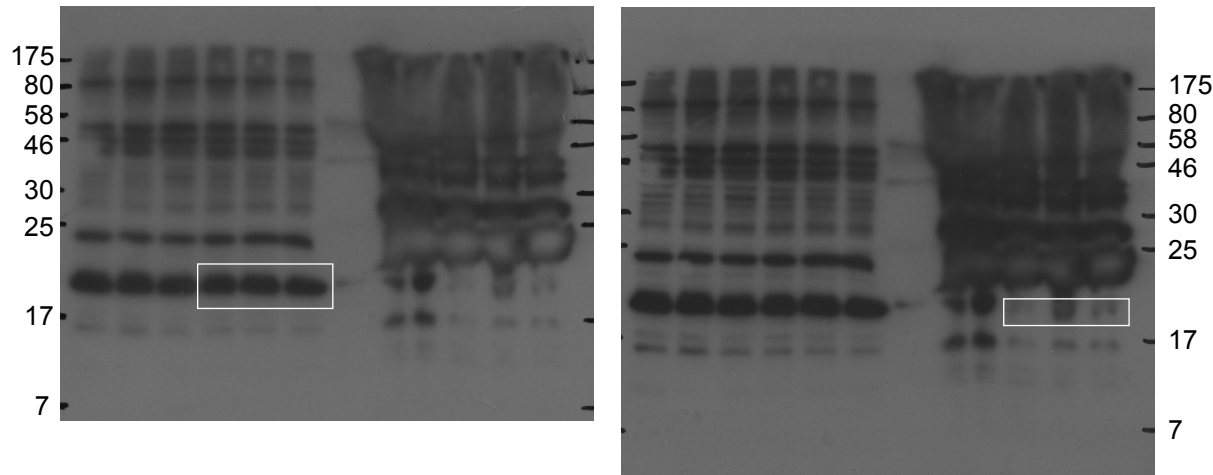

Panel B

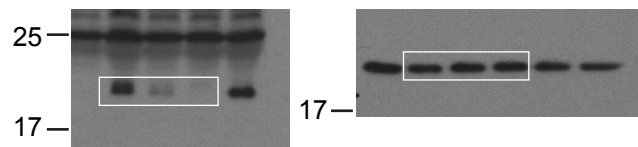

Supplement: Supplementary file 4 [file emmm0006-0239-sd4.pdf]

Giannotta et al., Figure S2

Panel B

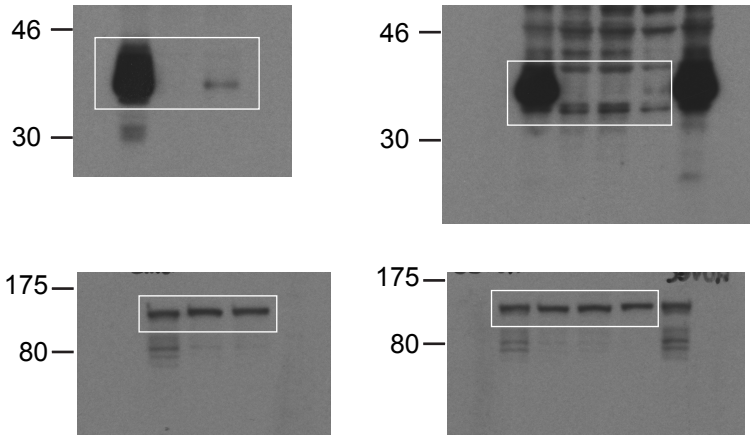

Supplement: Supplementary file 18 [file emmm0006-0239-sd18.pdf]

Panel A

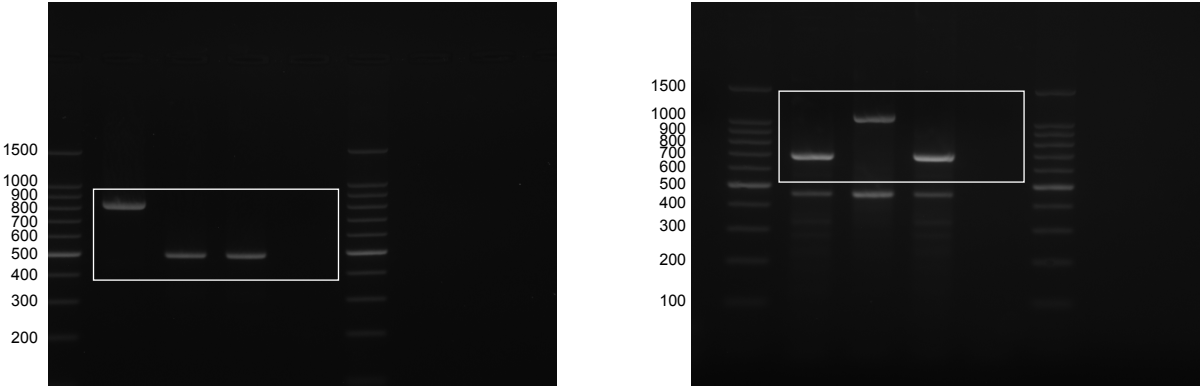

Supplement: Supplementary file 19 [file emmm0006-0239-sd19.pdf]

Panel B

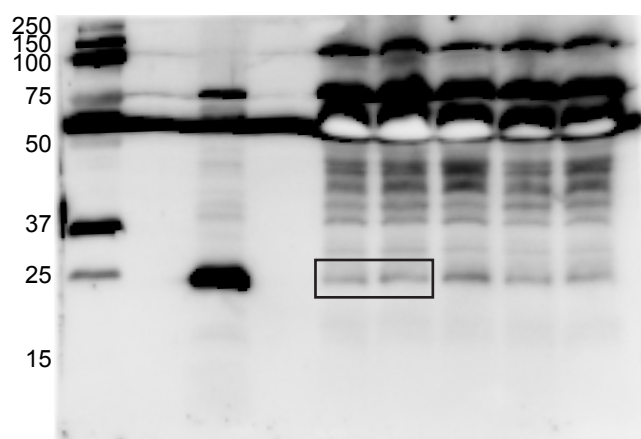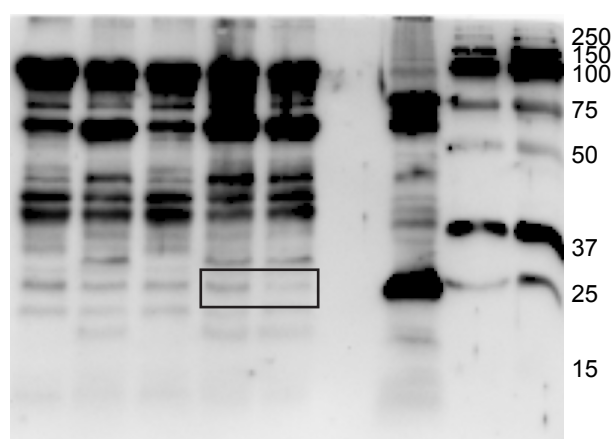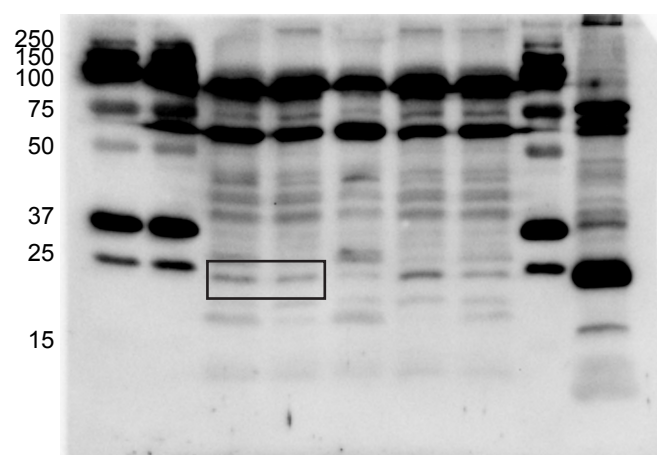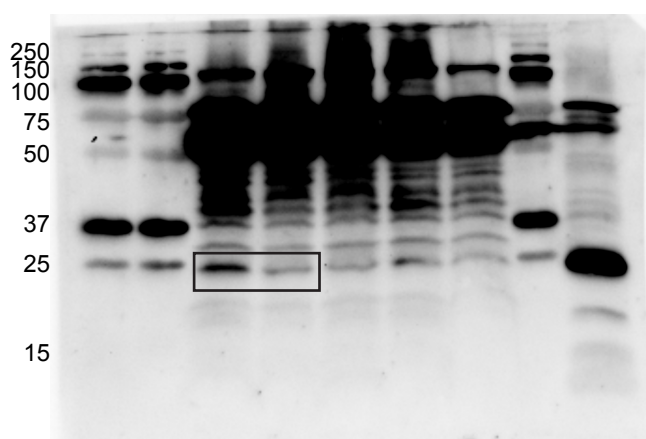

Supplement: Supplementary file 20 [file emmm0006-0239-sd20.pdf]

Panel B

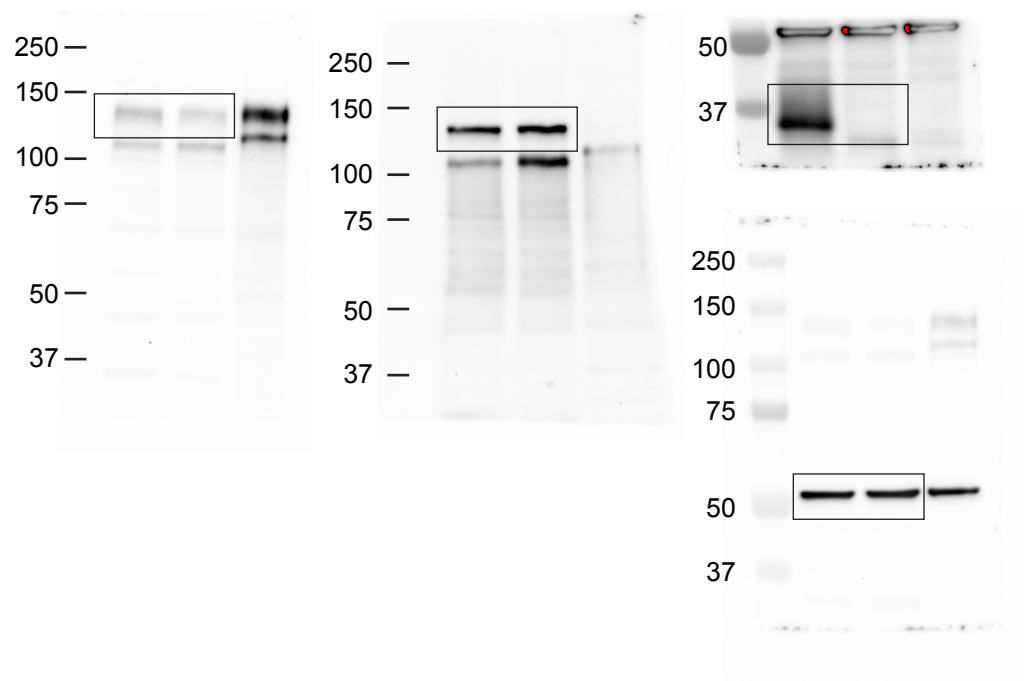

Supplement: Supplementary file 21 [file emmm0006-0239-sd21.pdf]
