# Supplementary material for: Targeting endothelial junctional adhesion molecule-A/ EPAC/ Rap-1 axis as a novel strategy to increase stem cell engraftment in dystrophic muscles
Source: EMBO Mol Med. 2013 Dec 30;6(2):239–58. doi: 10.1002/emmm.201302520 (PMC3927958; doi:10.1002/emmm.201302520)
Supplement: Supplementary file 5 [file emmm0006-0239-sd5.pdf]

Giannotta et. al, Figure S1

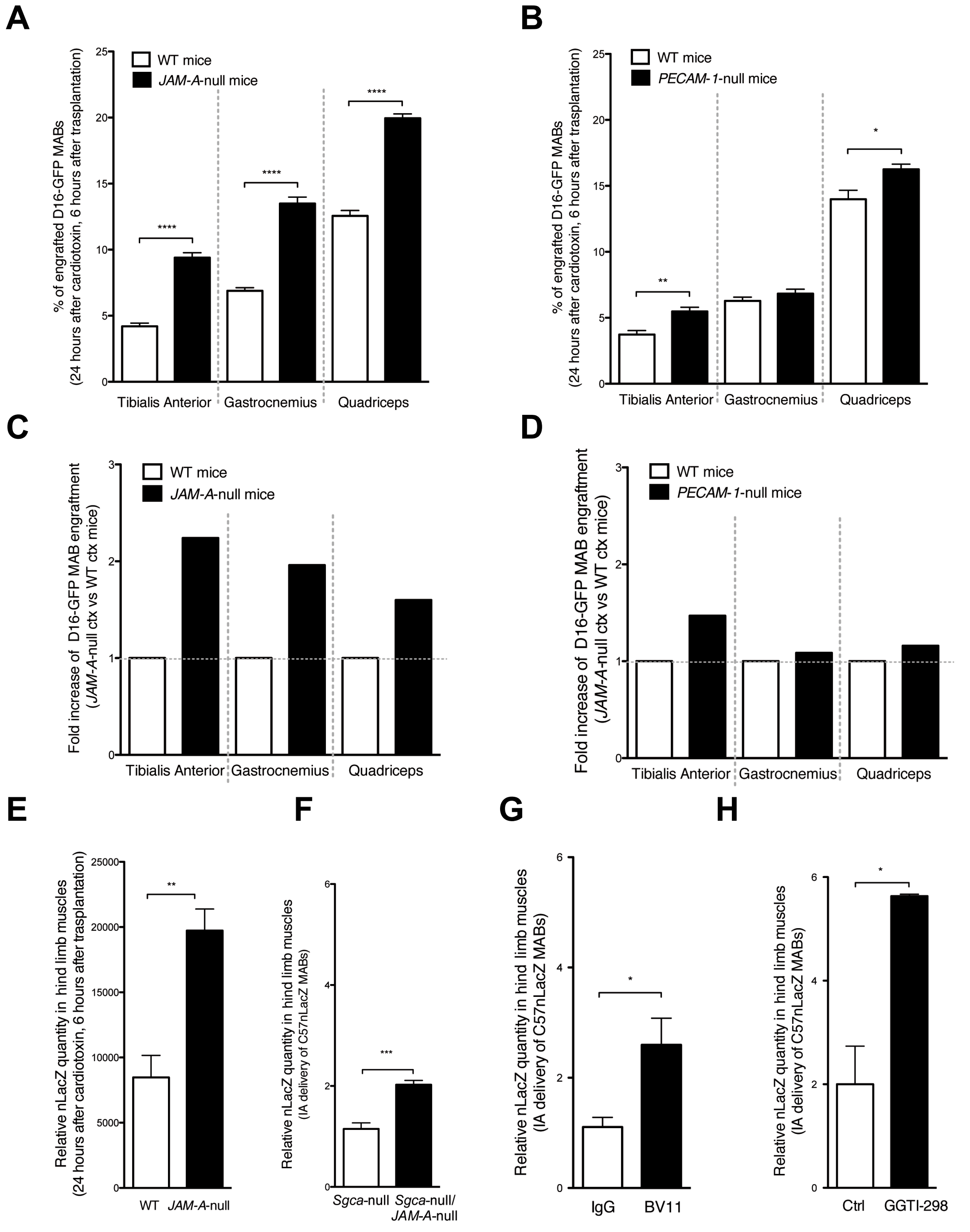

Figure S1. Engraftment of murine mesoangioblasts into acutely or chronically injured skeletal hind limb muscles. **A**. 2.2 x10^5 D16-GFP embryonic murine mesoangioblasts (MABs) were injected into the femoral artery of WT (n = 7) and *JAM-A*-null (n = 7) (treated with cardiotoxin 24 h before transplantation) as indicated in the legend. Plotted values indicated the percentage of engrafted cells per each analyzed muscle. The values have been calculated as the percentage of cells that reach each muscle compared to the total number of injected cells. **B**. 2.2 x10^5 D16-GFP embryonic murine MABs were injected into the femoral artery of WT (n = 7) and *PECAM-1*-null (n = 7) mice and the percentage of engrafted cells has been plotted. **C**. Fold increases of D16-GFP MABs engraftment in *JAM-A*-null mice versus WT mice. Controls (WT mice) have been set as 1 and data have been extrapolated from graph A. **D**. Fold increases of D16-GFP MAB engraftment in *PECAM-1*-null mice versus WT mice. Controls (WT mice) have been set as 1 and data have been obtained from ones shown in B. **E.** 10^6 C57-nLacZ adult murine mesoangioblasts (MABs) have been intra-arterially injected in WT (n = 3) and *JAM-A*-null (n = 3) mice 24 h after cardiotoxin treatment. Plotted values indicate the relative nLacZ levels. **F**. 10^6 C57-nLacZ MABs have been injected intra-arterially in *Sgca*-null and *Sgca*-null/JAM-A-null mice age-matched mice. Plotted values indicate the relative nLacZ levels. **G.** 10^6 C57-nLacZ MABs have been injected intra-arterially in *Sgca*-null treated with IgG (Ctr) and *Sgca*-null/ mice. Plotted values indicate the relative nLacZ levels. **H.** 10^6 C57-nLacZ MABs have been injected intra-arterially in *Sgca*-null treated with vehicle (Ctr) and *Sgca*-null mice treated with GGTI-298. Plotted values indicate the relative nLacZ levels. In all experiments hind limb muscles (tibialis anterior, gastrocnemius and quadriceps) were collected 6 h after intra-arterial delivery, and the presence of migrated cells was quantified using qRT-PCR with GFP primers. The RNA levels were normalized using GAPDH. Data are expressed as means ±SEM. * p <0.1, ** p <0.01, *** p <0.001, **** p <0.0001.
