# Supplementary material for: Targeting endothelial junctional adhesion molecule-A/ EPAC/ Rap-1 axis as a novel strategy to increase stem cell engraftment in dystrophic muscles
Source: EMBO Mol Med. 2013 Dec 30;6(2):239–58. doi: 10.1002/emmm.201302520 (PMC3927958; doi:10.1002/emmm.201302520)
Supplement: Supplementary file 6 [file emmm0006-0239-sd6.pdf]

Giannotta et al., Figure S2

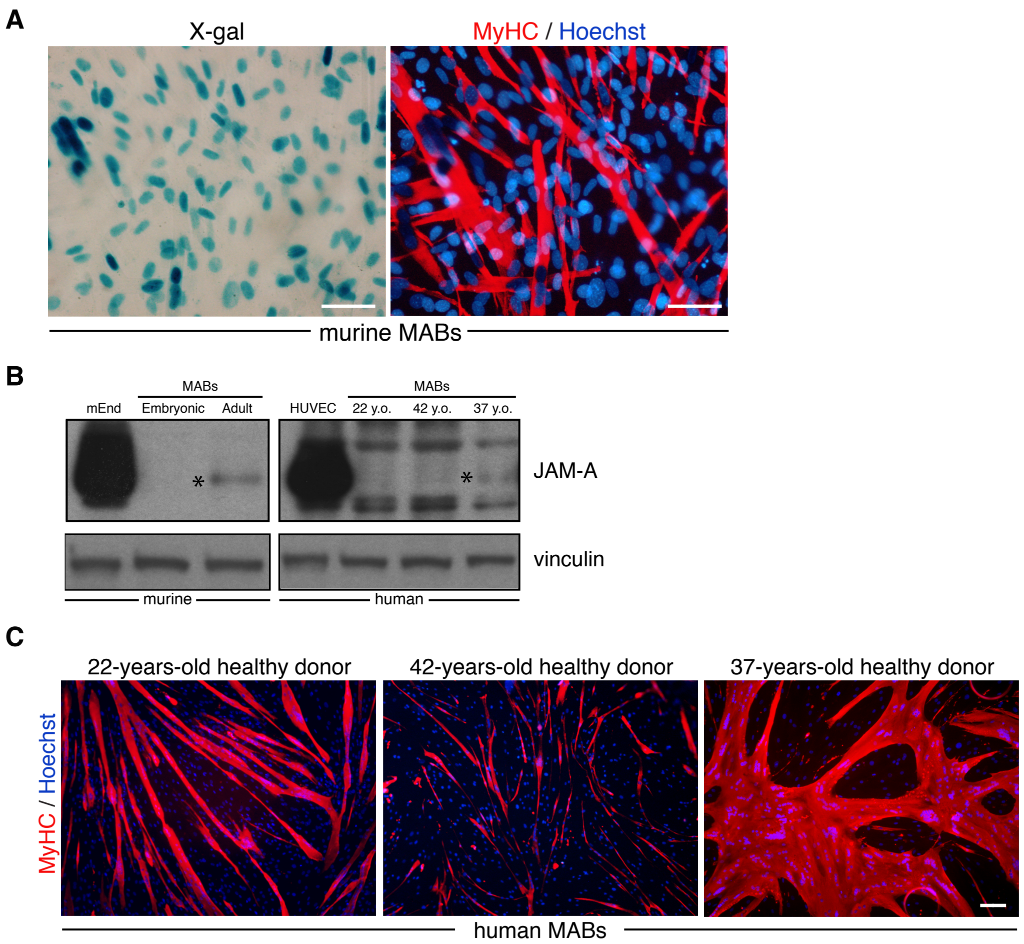

Figure S2. Spontaneous *in vitro* skeletal-muscle differentiation and endogenous JAM-A expression in murine and human mesoangioblasts. A. C57 adult murine MABs previously transduced with a lentiviral vector expressing a nuclear LacZ (C57-nLacZ) were fixed and stained with X-gal (left), myosin heavy chain (MyHC, right: red) and Hoechst (right: blue). Merged images of red and blue signals are shown (right). **B**. Both embryonic and adult murine MABs (left panels), as well as human MABs derived from three different healthy donors (22, 42 and 37 y.o.) (right panels) were homogenised. The cell lysates were analysed by immunoblotting for murine and human JAM-A (asterisks) using vinculin as loading control. Murine (mEnd) (left panel) and human (HUVEC) endothelial cells (right panel) were used as a positive control. **C**. Human MABs with different myogenic potentials, as derived from three healthy donors (as indicated). The cells were fixed and stained for MyHC (red) and Hoechst (blue), with merged images shown. Scale bars: 50 μm.
