# Supplementary material for: Targeting endothelial junctional adhesion molecule-A/ EPAC/ Rap-1 axis as a novel strategy to increase stem cell engraftment in dystrophic muscles
Source: EMBO Mol Med. 2013 Dec 30;6(2):239–58. doi: 10.1002/emmm.201302520 (PMC3927958; doi:10.1002/emmm.201302520)
Supplement: Supplementary file 7 [file emmm0006-0239-sd7.pdf]

Giannotta et al., Figure S3

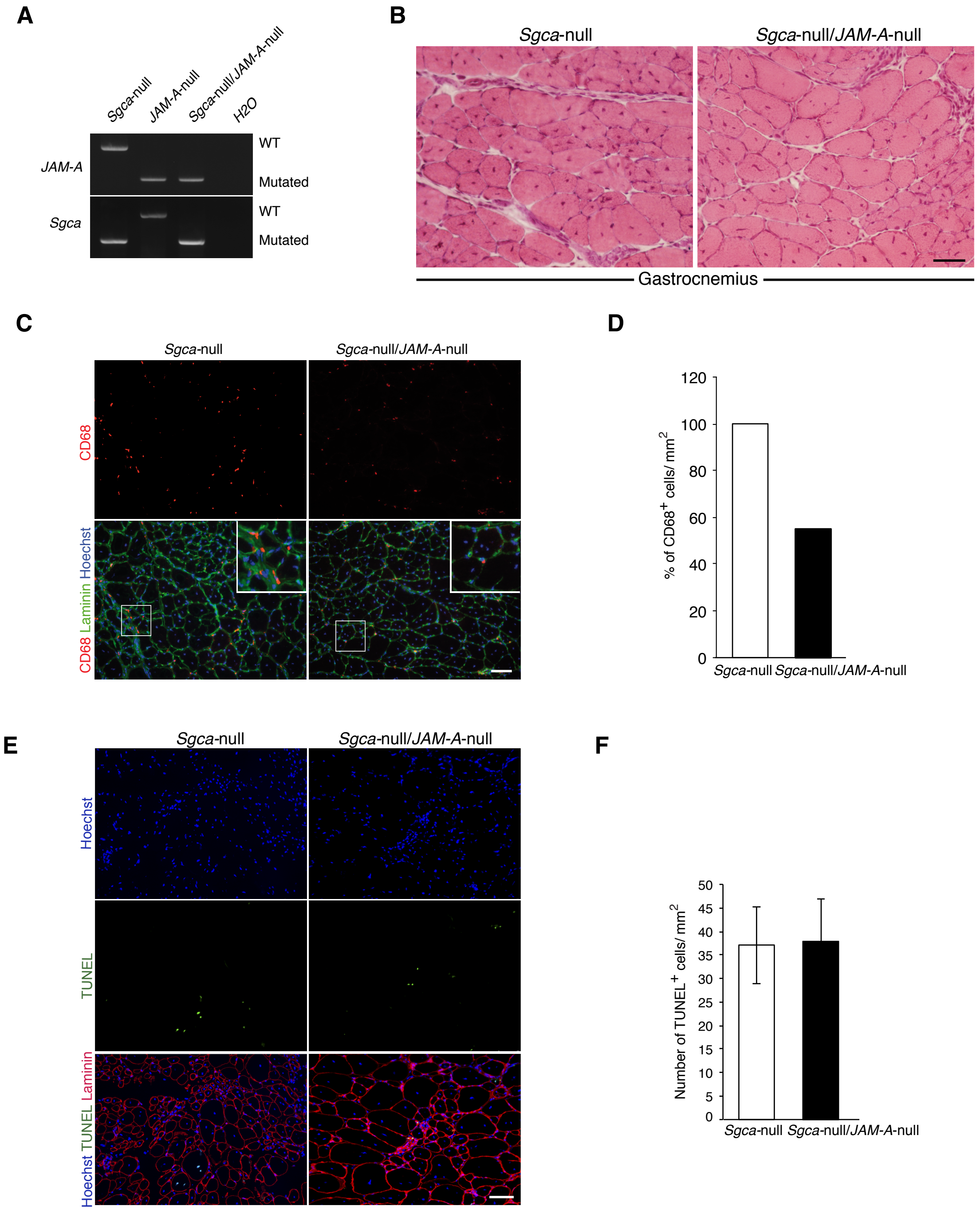

Figure S3. Characterization of *Sgca*-null/*JAM-A* null mice compared to *Sgca*-null mice. A. Genotyping of *Sgca*-null, *JAM-A*-null and *Sgca*-null/*JAM-A*-null mice. For *JAM-A* gene: WT allele, 800 bp; mutated allele, 500 bp. For *Sgca* gene: WT allele, 1066 bp; and mutated allele, 618 bp. **B.** Hematoxylin and eosin staining of transversal skeletal muscle cryosections of *Sgca*-null and *Sgca*-null/*JAM-A*-null mice. **C.** Representative transversal skeletal muscle cryosections from *Sgca*-null (left) and *Sgca*-null/*JAM-A*-null (right) mice stained for $CD68^+$ macrophages (top, red) laminin (bottom, green) and Hoechst (bottom, blue). Merged images of red, green and blue signals are shown (bottom). Insets: high magnification showing clusters of $CD68^+$ macrophages in muscles of *Sgca*-null and *Sgca*-null/*JAM-A*-null mice. **D.** Quantification of $CD68^+$ macrophages shown in C. $CD68^+$ cells are expressed as percentages of cells per area. **E.** TUNEL staining (green, middle row) of transversal skeletal muscle cryosections from *Sgca*-null (left column) and *Sgca*-null/*JAM-A*-null (right column) mice. Sections have been counterstained for laminin (bottom row, red) and Hoechst (top row, blue). Merged images of blue, green and red signals are shown (bottom row). **F.** Quantification of $TUNEL^+$ cells/mm^2 in *Sgca*-null and *Sgca*-null/*JAM-A*-null mice. $TUNEL^+$ cells are expressed as number of positive cells per area. Scale bar: 100 μm. Data are means ±SEM.
