# Supplementary material for: Targeting endothelial junctional adhesion molecule-A/ EPAC/ Rap-1 axis as a novel strategy to increase stem cell engraftment in dystrophic muscles
Source: EMBO Mol Med. 2013 Dec 30;6(2):239–58. doi: 10.1002/emmm.201302520 (PMC3927958; doi:10.1002/emmm.201302520)
Supplement: Supplementary file 8 [file emmm0006-0239-sd8.pdf]

Giannotta et al., Figure S4

A

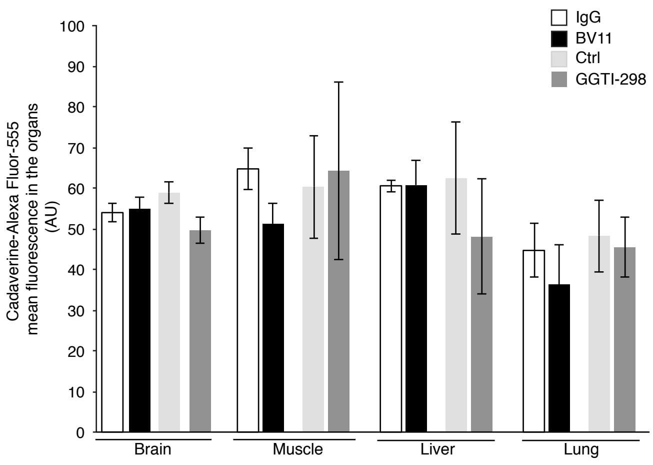

B

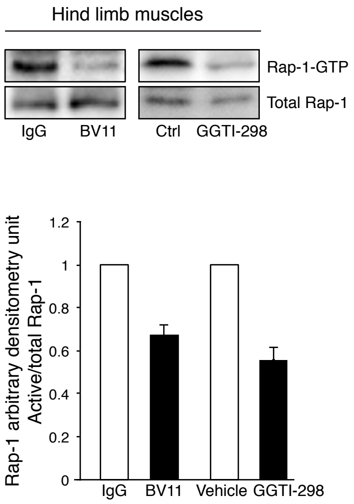

Figure S4. BV11 antibody and GGTI-298 treatments inhibit Rap-1 activation in skeletal muscle of *Sgca*-null mice and do not induce tissue edema. A. *Sgca*-null mice were treated with an anti-JAM-A neutralizing antibody (BV11, 3 mg/kg, black columns, n =3) or with IgG (IgG, 3 mg/kg, white columns, n = 3) as control. After 30 min, the mice were injected with cadaverine-Alexa Fluor-555 (25 mg/kg) and 2 h later they were sacrificed and their organs were collected. Whole brains, lungs, livers and muscle were photographed and cadaverine was quantified. The presence of cadaverine in the organs was expressed in arbitrary units as mean fluorescence. Similarly, *Sgca*-null mice were treated with GGTI-298 (GGTI-298, dark grey columns, n = 3) or with vehicle (Ctrl, light grey columns, n = 3) as control. Mice have been treated as described above. **B.** *Sgca*-null mice were treated as in A. After 2 h, the hind limb muscles (gastrocnemius, tibialis anterior and quadriceps) were collected and then processed by Tissue Lyser II. The protein extracts were incubated with the Ral-GDS-RBD probe. The active Rap-1 (Rap-1-GTP) and total Rap-1 were detected using an anti-Rap-1 antibody. Representative data are shown for the densitometry analysis of active Rap-1-GTP, normalized for total protein. Data are means ±SD from three independent experiments (n = 3 for each condition).
