# Supplementary material for: Targeting endothelial junctional adhesion molecule-A/ EPAC/ Rap-1 axis as a novel strategy to increase stem cell engraftment in dystrophic muscles
Source: EMBO Mol Med. 2013 Dec 30;6(2):239–58. doi: 10.1002/emmm.201302520 (PMC3927958; doi:10.1002/emmm.201302520)
Supplement: Supplementary file 12 [file emmm0006-0239-sd12.pdf]

Giannotta et al., Figure S5

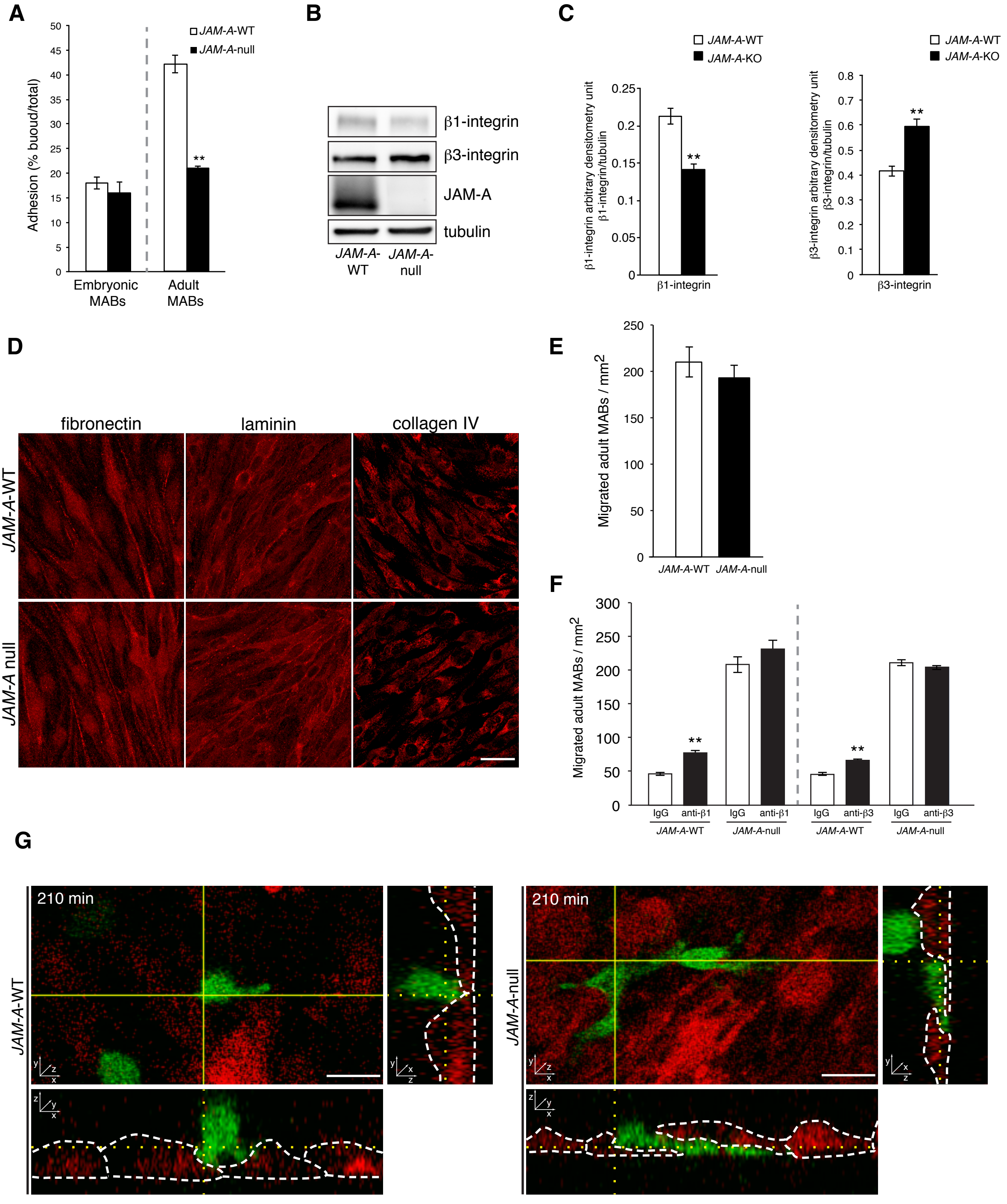

Figure S5. Adhesion, β1- β3-integrins activation and the components of the extracellular matrix are not responsible for the increase in mesoangioblast transmigration through *JAM-A*-null endothelial cells

A. Adhesion of embryonic and adult MABs to *JAM-A*-WT and *JAM-A*-null endothelial cells was performed as described in the Materials and Methods section. Data are expressed as percentage of total MABs and are means ±SEM from three independent experiments, each in triplicate. ** $p < 0.001$ *vs* *JAM-A*-WT endothelial cells. **B.** Cultured endothelial cells isolated from *JAM-A*-WT and *JAM-A*-null mice were homogenized. The lysates were analyzed by immunoblotting for β1-, β3-integrins and JAM-A using tubulin as a loading control. **C.** Densitometry analysis of β1- β3-integrins expression level, normalized for tubulin. ** $p < 0.001$ *vs* *JAM-A*-WT endothelial cells. Data are means ±SD from three independent experiments. **D.** Confluent monolayers of *JAM-A*-WT and *JAM-A*-null endothelial cells were fixed and stained with anti-fibronectin, anti-laminin, anti collagen IV. Scale bar: 50 μm. **E.** *JAM-A*-WT and *JAM-A*-null endothelial cells were seeded onto glutaraldehyde-crosslinked gelatin-coated filters for 72 h. Following endothelium removal (as described in the Methods section), 6-CFDA-labeled adult MABs were added to the upper chamber and allowed to migrate for 3 h. The migrated MABs on the lower sides of the filters were fixed and counted. Quantification of the number of migrated MABs per area is shown. Data are means ±SEM from three independent experiments, each in triplicate. **F.** *JAM-A*-WT and *JAM-A*-null endothelial cells were seeded onto glutaraldehyde-crosslinked gelatin-coated filters for 72 h. Then, the cells were pre-treated (3 h) and further incubated (6 h) with non-related IgG (10 μg/ml or 3 μg/ml), β1-integrin (10 μg/ml), β3-integrin (3 μg/ml) blocking antibodies, as indicated. Quantification of the number of migrated MABs per area. ** $p < 0.001$ *vs* non related IgG. Data are means ±SEM from four independent experiments, each in triplicate. **G.** Time-lapse imaging of adult MAB (C57-GFP, green) transmigration across *JAM-A*-WT (left, red) and *JAM-A*-null (right, red) endothelial cells expressing Td-tomato seeded onto collagen matrix. Images were obtained every 7 min from 0 (time of MAB addition to the endothelial monolayers) to 380 min. Orthogonal cross-sections of a representative confocal image of MAB transmigration after 210 min are shown. Scale bar: 20 μm.
