# Supplementary material for: Targeting endothelial junctional adhesion molecule-A/ EPAC/ Rap-1 axis as a novel strategy to increase stem cell engraftment in dystrophic muscles
Source: EMBO Mol Med. 2013 Dec 30;6(2):239–58. doi: 10.1002/emmm.201302520 (PMC3927958; doi:10.1002/emmm.201302520)
Supplement: Supplementary file 13 [file emmm0006-0239-sd13.pdf]

Giannotta et al., Figure S6

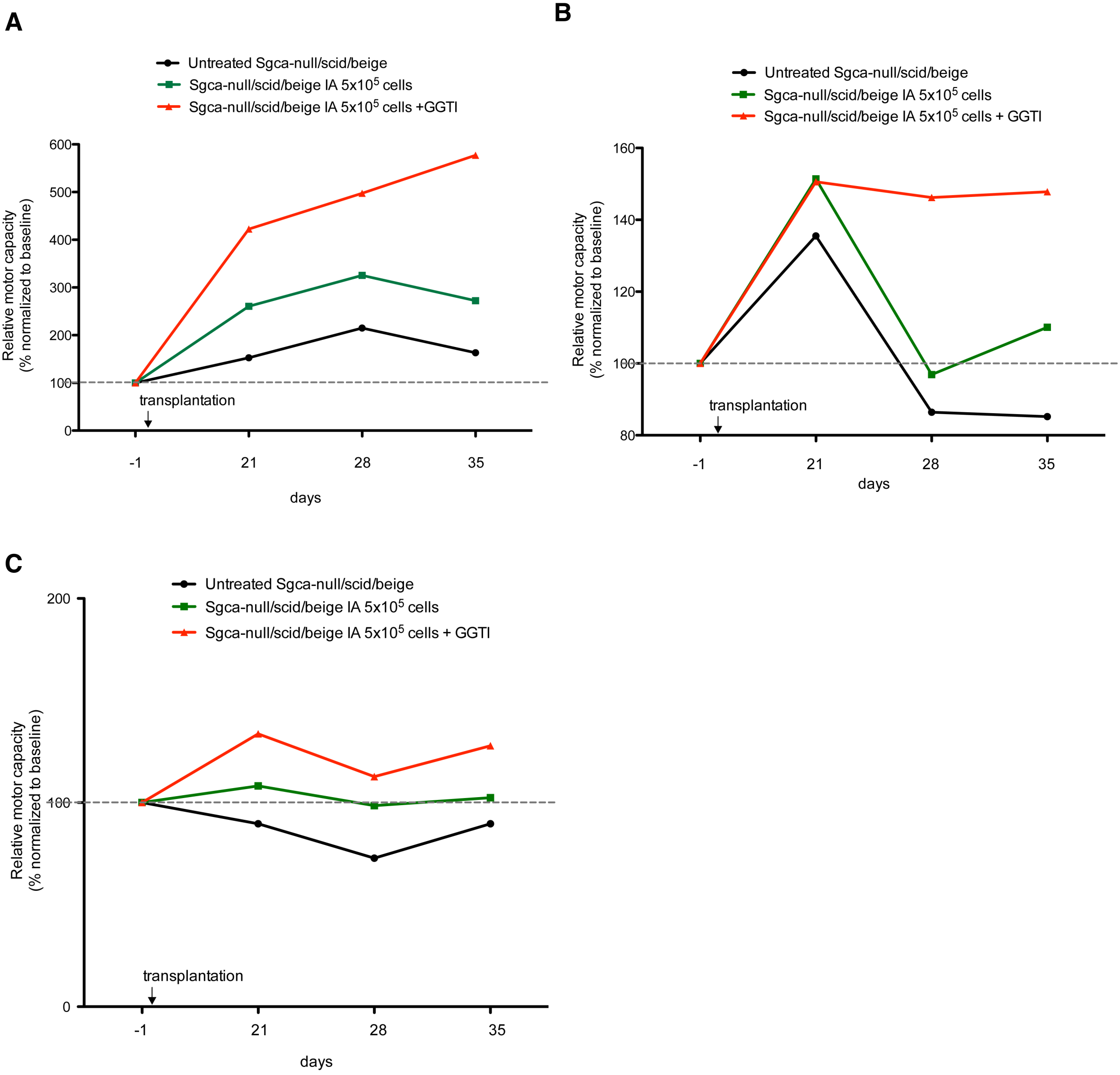

Figure S6. Time to exhaustion in a treadmill test. **A**. Trend of relative motor capacity for age- and sex-matched 2 months old mice: untransplanted *Sgca*-null/scid/beige, transplanted *Sgca*-null/scid/beige and transplanted *Sgca*-null/scid/beige treated with GGTI-298 (n = 3). **B**. Trend of relative motor capacity for age- and sex-matched 4 months old mice: *Sgca*-null/ scid/beige, transplanted *Sgca*-null/ scid/beige and transplanted *Sgca*-null/scid/beige treated with GGTI-298 (n = 3). **C**. Trend of relative motor capacity for age- and sex-matched 7 months old mice: *Sgca*-null/scid/beige, transplanted *Sgca*-null/ scid/beige and transplanted *Sgca*-null/ scid/beige treated with GGTI-298 (n = 3). Untransplanted *Sgca*-null/scid/beige: bilateral injection with PBS (vehicle); transplanted *Sgca*-null/scid/beige: $5x10^5$ cells bilateral injection via femoral artery; transplanted *Sgca*-null/scid/beige treated with GGTI-298: treatment with GGTI-298 1 h prior $5x10^5$ cells bilateral injection via femoral artery.
