# Supplementary material for: Targeting endothelial junctional adhesion molecule-A/ EPAC/ Rap-1 axis as a novel strategy to increase stem cell engraftment in dystrophic muscles
Source: EMBO Mol Med. 2013 Dec 30;6(2):239–58. doi: 10.1002/emmm.201302520 (PMC3927958; doi:10.1002/emmm.201302520)
Supplement: Supplementary file 14 [file emmm0006-0239-sd14.pdf]

Table S1. List of primers used in this study

**Genotyping PCR primers**	**Primer sequence (5'-3')**		**Product size (bp)**
Sgca	INT1: CAGGGCTGGGAGCTGGGTTCTG<br>EX2 : CCCAGGGCCTTGATGCCT<br>NEOTR: GCTATCAGGACATAGCGTTGGCTA		1066 bp WT allele<br>618 bp mutated allele
JAM-A	BV379: CTTTTCACCAATCGGAACGCG<br>BV512: TTCATTTCTTCTTCAGACG		800 bp WT allele<br>600 bp mutated allele
**qRT-PCR primers Gene name**	**Primer sequence (5'-3')**		**Product size (bp)**
nLacZ	FW: ATCTCTATCGTGCGGTGGTT	REV: GAGCTGACCATGCAGAGGAT	175 bp
GFP	FW: ACAAGCAGAAGAACGGCATC	REV: CGGTCACGAACTCCAGCA	213 bp
GAPDH	FW: TTCACCACCATGGAGAAGGC	REV: GGCATGGACTGTGGTCATGA	250 bp
**shRNA target name**	**shRNA sequence**		
JAM-A	shRNA#49: CCGGCCAGACTCGTTTGCTATAATACTCGAGTATTATAGCAAACGAGTCTGGTTTTTG<br>shRNA#50: CCGGCCACTTTGACAGAACAAAGAACTCGAGTTCTTTGTTCTGTCAAAGTGGTTTTTG<br>shRNA#51: CCGGGCAGTGTTACAGTGCACTCTTCTCGAGAAGAGTGCACTGTAACACTGCTTTTTG<br>shRNA#52: CCGGGCCAACTGGTATCACCTTCAACTCGAGTTGAAGGTGATACCAGTTGGCTTTTTG		
non-targeting shRNA	shRNA: CCGGCAACAAGATGAAGAGCACCAACTCGAGTTGGTGCTCTTCATCTTGTTGTTTTT		
**Cloning PCR primers**	**Primer sequence (5'-3')**		**Product size (bp)**
PECAM-1	FW: AGCTTTCTTCGAATGCTCCTGGCTCTGGGACTC<br>RW: TCCCCCCGGGAAGTTCCATTAAGGGAGCCTTC		2000 bp

FW, forward; RW, reverse.
