# Supplementary material for: Targeting endothelial junctional adhesion molecule-A/ EPAC/ Rap-1 axis as a novel strategy to increase stem cell engraftment in dystrophic muscles
Source: EMBO Mol Med. 2013 Dec 30;6(2):239–58. doi: 10.1002/emmm.201302520 (PMC3927958; doi:10.1002/emmm.201302520)
Supplement: Supplementary file 15 [file emmm0006-0239-sd15.pdf]

Table S2. List of antibodies used in this study

Antibody	Assay	Company	Dilution
Rabbit anti-β-gal	IF	Cappel	1:300
Mouse anti-Sgca	IF	Novocastra	1:50
Mouse anti-MyHC MF20	IF	Developmental Studies Hybridoma Bank	1:2
Chicken anti-laminin	IF	Abcam	1:500
Rabbit anti-laminin	IF	Sigma-Aldrich	1:300
Rabbit anti-GST	IF	Abcam	1:400
Rat anti-mouse-VE-cadherin	IF	BD Biosciences	1:100
Goat anti-VE-cadherin	IF, WB	Santa Cruz Biotechnology	1:200, 1:500
Mouse anti-vinculin	WB	Sigma-Aldrich	1:2000
Rat anti-mouse JAM-A (BV20)	WB	Home-made	5 μg/ml
Rat anti-JAM-A (BV11)	BL	Home-made	20 μg/ml
Mouse anti-JAM-A (BV16)	BL	Home-made	12 μg/ml
Goat anti-mouse JAM-A	IF, WB	R&D	1:200, 1:2500
Rat anti-mouse JAM-A H2O2-106	IP	Santa Cruz Biotechnology	4 μg/mg protein
Rabbit anti-human JAM-A	IF	Invitrogen	1:100
Mouse anti-EPAC-1(5D3)	WB	Cell Signaling Technology	1:1000
Mouse anti-EPAC-2 (5B1)	WB	Cell Signaling Technology	1:500
Rabbit anti-EPAC-2 (H-220)	IP	Santa Cruz Biotechnology	4 μg/mg protein
Rabbit anti-Rap-1(121)	WB	Santa Cruz Biotechnology	1:200
Mouse anti-GAPDH (6C5)	WB	Santa Cruz Biotechnology	1:1000
Mouse IgG isotype (MOPC 21)	BL control	Sigma-Aldrich	12 μg/ml
Rat IgG isotype	BL control	Sigma-Aldrich	20 μg/ml
Purified NA/LE Hamster anti-Mouse CD29	BL	BD Biosciences	10 μg/ml
Purified NA/LE Hamster IgG2, λ1	BL control	BD Biosciences	10 μg/ml
Purified NA/LE Hamster anti-Mouse CD61	BL	BD Biosciences	3 μg/ml
Purified NA/LE Hamster IgG1, κ1	BL control	BD Biosciences	3 μg/ml
Rabbit anti-β1 integrin	WB	Cell Signaling Technology	1:1000
Rabbit anti-β3 integrin	WB	Cell Signaling Technology	1:1000
Chicken anti-laminin	IF	Abcam	1:500
Rabbit anti-collagen IV	IF	Serotec	1:200
Hamster anti-CD31	IF	Millipore	1:400
anti-fibronectin	IF	Abcam	1:50

Abbreviations: WB, Western blot; IF, immunofluorescence; BL, blocking; IP, immunoprecipitation
