# Supplementary material for: Targeting endothelial junctional adhesion molecule-A/ EPAC/ Rap-1 axis as a novel strategy to increase stem cell engraftment in dystrophic muscles
Source: EMBO Mol Med. 2013 Dec 30;6(2):239–58. doi: 10.1002/emmm.201302520 (PMC3927958; doi:10.1002/emmm.201302520)
Supplement: Supplementary file 16 [file emmm0006-0239-sd16.pdf]

Supporting Information

Figure S1. Engraftment of murine mesoangioblasts into acutely or chronically injured skeletal hind limb muscles……………………………..3

Legend Figure S1 ……………………………………………………….4

Figure S2. Spontaneous *in vitro* skeletal-muscle differentiation and endogenous JAM-A and Rap1 expression in murine and human mesoangioblasts……………………………………………………………5

Legend Figure S2 ……………………………………………………….6

Figure S3. Characterization of *Sgca*-null/*JAM-A* null mice compared to *Sgca*-null mice………………………………………………………………7

Legend Figure S3 ……………………………………………………….8

Figure S4. BV11 antibody and GGTI-298 treatments inhibit Rap-1 activation in skeletal muscle of *Sgca*-null mice and do not induce tissue edema……….9

Legend Figure S4 ……………………………………………………10

Figure S5. Adhesion, β1- β3-integrins activation and the components of the extracellular matrix are not responsible for the increase in mesoangioblast transmigration through *JAM-A*-null endothelial cells.................................11

Legend Figure S5 ………………………………………………………..12

Figure S6. Time to exhaustion in a treadmill test…………………………13

Legend Figure S6 ………………………………………………………..14

Table S1. List of primers used in this study…………………………………15

Table S2. List of antibodies used in this study………………………………16

Movie M1. Time-lapse imaging of adult mesoangioblast (C57-GFP, green) transmigration across *JAM-A*-WT (left, red) and *JAM-A*-null (right, red) endothelial cells expressing Td-tomato seeded onto collagen matrix………17

Movie M2. Time-lapse imaging of *JAM-A*-WT (left) and *JAM-A*-null (right) endothelial cells expressing PECAM-1-GFP as marker of cell-cell junction (white)……………………………………………………………………17
Movie M3. Time-lapse imaging of C57-GFP mesoangioblast transmigration across and *JAM-A*-null endothelial cells expressing PECAM-1-GFP (white) seeded onto collagen matrix……………………..17
