# Supplementary material for: Targeting endothelial junctional adhesion molecule-A/ EPAC/ Rap-1 axis as a novel strategy to increase stem cell engraftment in dystrophic muscles
Source: EMBO Mol Med. 2013 Dec 30;6(2):239–58. doi: 10.1002/emmm.201302520 (PMC3927958; doi:10.1002/emmm.201302520)
Supplement: Supplementary file 17 [file emmm0006-0239-sd17.pdf]

Movie M1. Time-lapse imaging of adult mesoangioblast (C57-GFP, green) transmigration across *JAM-A*-WT (left, red) and *JAM-A*-null (right, red) endothelial cells expressing Td-tomato seeded onto collagen matrix. Images stack were obtained every 7 min from 0 (time of mesoangioblast addition to the endothelial monolayers) to 380 min. Stack maximum projection along *z*-axis (upper panels) and *y*-axis (lower panels) are shown. The video speed is 5 frames/second. Representative frames of this movie are shown in Fig 5A and Fig S5G.

Movie M2. Time-lapse imaging of *JAM-A*-WT (left) and *JAM-A*-null (right) endothelial cells expressing PECAM-1-GFP as marker of cell-cell junction (white). Images stack were obtained every 12 min for 300 min. Stack maximum projection along *z*-axis are shown. The video speed is 5 frames/second. Representative frames of this movie are shown in Fig 5B.

Movie M3. Time-lapse imaging of C57-GFP mesoangioblast transmigration across *JAM-A*-null endothelial cells expressing PECAM-1-GFP (white) seeded onto collagen matrix. Images stack were obtained every 10 min for 300 min. Stack maximum projection along *z*-axis are shown. Mesoangioblast is shown as a gradient of pseudo-colors (Depth index) ranging from red (top, on the endothelium) to blue (bottom, through the collagen matrix), varying according to focal plane depth. The video speed is 5 frames/second. Representative frames of this movie are shown in Fig 5C.
